# A single-cell comparison of adult and fetal human epicardium defines the age-associated changes in epicardial activity

**DOI:** 10.1038/s44161-022-00183-w

**Published:** 2022-12-21

**Authors:** Vincent R. Knight-Schrijver, Hongorzul Davaapil, Semih Bayraktar, Alexander D. B. Ross, Kazumasa Kanemaru, James Cranley, Monika Dabrowska, Minal Patel, Krzysztof Polanski, Xiaoling He, Ludovic Vallier, Sarah Teichmann, Laure Gambardella, Sanjay Sinha

**Affiliations:** 1 Wellcome-MRC Cambridge Stem Cell Institute, Jeffrey Cheah Biomedical Centre, Cambridge Biomedical Campus, University of Cambridge, Cambridge, UK; 2 Department of Paediatrics, University of Cambridge, Cambridge, UK; 3 Department of Medical Genetics, University of Cambridge, Cambridge, UK; 4 Wellcome Sanger Institute, Wellcome Genome Campus, Cambridge, UK; 5 John van Geest Centre for Brain Repair, Cambridge University, Cambridge, UK; 6 Berlin Institute of Health (BIH), BIH Centre for Regenerative Therapies (BCRT), Charité - Universitätsmedizin, Berlin, Germany; 7 Max Planck Institute for Molecular Genetics, Berlin, Germany; 8 Cavendish Laboratory, Department of Physics, University of Cambridge, Cambridge, UK; 9 These authors jointly supervised this work: Laure Gambardella, Sanjay Sinha

## Abstract

Re-activating quiescent adult epicardium represents a potential therapeutic approach for human cardiac regeneration. However, the exact molecular differences between inactive adult and active fetal epicardium are not known. In this study, we combined fetal and adult human hearts using single-cell and single-nuclei RNA sequencing and compared epicardial cells from both stages. We found that a migratory fibroblast-like epicardial population only in the fetal heart and fetal epicardium expressed angiogenic gene programs, whereas the adult epicardium was solely mesothelial and immune responsive. Furthermore, we predicted that adult hearts may still receive fetal epicardial paracrine communication, including WNT signaling with endocardium, reinforcing the validity of regenerative strategies that administer or reactivate epicardial cells in situ. Finally, we explained graft efficacy of our human embryonic stem-cell-derived epicardium model by noting its similarity to human fetal epicardium. Overall, our study defines epicardial programs of regenerative angiogenesis absent in adult hearts, contextualizes animal studies and defines epicardial states required for effective human heart regeneration.

A major challenge to human health is that the adult human heart does not regenerate. Myocardial infarction (MI) causes a permanent non-contractile and non-conductive scar, which leads to chronic heart failure and arrhythmia. Much interest has followed the epicardium recently for its key role in heart development and potential to contribute to heart regeneration.

The epicardium emerges from the proepicardium during cardiogenesis as a mesothelial layer of cells surrounding the heart^1^. During development, epicardial cells may lose mesothelial identity and undergo epithelial-to-mesenchymal transition (EMT), resulting in a population of epicardial-derived cells (EPDCs) that migrate into the myocardium^2^. These EPDCs may differentiate into smooth muscle cells, cardiac fibroblasts and, potentially, endothelial cells^3,4^. Furthermore, developing epicardial cells and EPDCs secrete potent factors, including WNT, FGFs and PDGFs, which stimulate vasculogenesis and the proliferation and maturation of cells within the myocardial tissue^5^. These develop-mental abilities also translate into a regenerative role. Adult zebrafish hearts are capable of regeneration, and developmental epicardial genes become highly expressed at the infarcted region, coinciding with the restoration of cardiac muscle^6,7^. Likewise, when cardiac regeneration is seen in embryos and neonates of small and large mammals, including humans^8–10^, the active epicardium responds with EMT and the secretion of angiogenic factors^11,12^. However, these studies also illustrate that any regenerative window in mammals soon disappears after birth.

In contrast, the adult mammalian epicardium is normally quiescent, with reduced secretory and migratory capacities, and, although it appears to reactivate after injury, the response may not be strong or rapid enough for sufficient regeneration^13^. However, there is evidence that a properly active epicardium can still promote regeneration of adult mammalian hearts; studies have established the efficacy and essentiality of epicardial-directed repair mechanisms, such as thymosin-β-4, FGFs and even exosome-mediated signaling in successful cardiac regeneration^14–16^. Additionally, human embryonic stem cell (hESC)-derived epicardium (hESC-EPI), when administered alongside hESC-derived cardiomyocytes, increases vascularization, proliferation and survival of myocardial tissue^17^.

Altogether, the evidence suggests that the epicardium augments heart regeneration and that timely reactivation of epicardial programs offers a promising therapeutic strategy for treating MI in humans. However, without an in-depth understanding of the epicardium in humans, our ability to translate these models into a therapeutic context is limited. As it stands, it is still not fully known if the adult human epicardium retains gene expression from development or if its response to injury is similar to that seen in animals or how it relates to hESC-EPI. Additionally, epicardial cells across many species and humans are identified using *WT1*, *TBX18* and *TCF21* (refs. 18,19) but can be further divided into heterogeneous subpopulations in zebrafish and in hESC-EPI^20,21^. Additionally, adult human epicardium may be identified through its coexpression of *BNC1* and *MSLN*^22^. However, epicardial heterogeneity may not occur clearly in mammals in vivo^23^ and has not been fully explored in humans of any age. Therefore, in light of this missing knowledge, we attempted to define the key factors of epicardial-derived regeneration that are lost in adults and aimed to capture the different transcriptional states of human epicardium, define age-associated changes in epicardial populations and reveal distinct signaling pathways that are associated with fetal or adult epicardium.

We addressed our aims using single-cell RNA sequencing (scRNA-seq) to isolate epicardial cells in silico and mitigate biases from sorting and selection. Although scRNA-seq data of both adult and fetal hearts have been generated and analyzed independently, no attempt has been made to combine them^22,24^. We integrated adult and fetal human hearts at a single-cell resolution, which allowed us to compare the epicardium in both stages. We approached our dataset from multiple angles and triangulated the epicardium in both adult and fetal cells using prior knowledge and unbiased clustering of datasets, both mixed and separated. This approach converged on a detailed profile of human epicardial cells, allowing us to (1) identify fetal epicardial subtypes, (2) create a library of epicardial markers for translating animal studies, (3) reveal an angiogenic program of epicardial communication not present in adult humans and (4) validate hESC-EPIs as a model of human fetal epicardium.

## Results

### Two stages of the human heart are integrated

**Extended Data Fig. 1:**
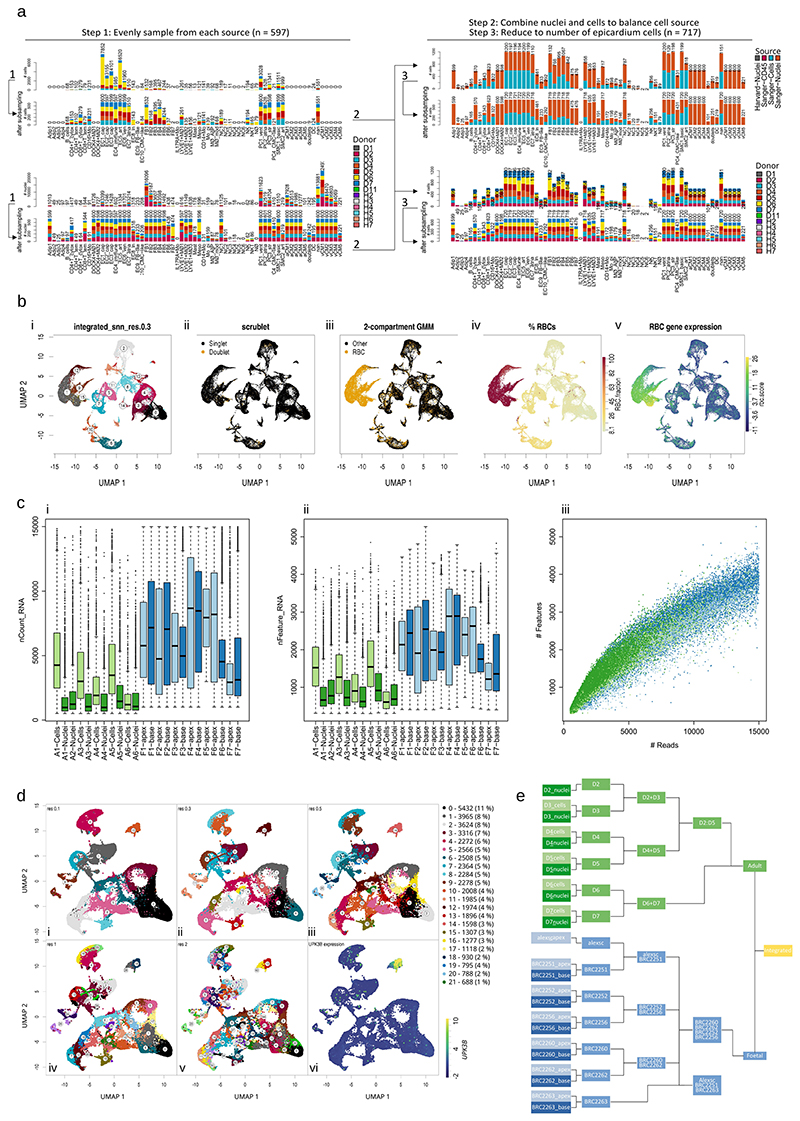
Stratified sampling, integration, and quality of adult and fetal scRNA-seq datasets. Processing of adult and fetal datasets and integration with **a**, subsampling stratifications and step-wise subsampling strategy for adult cells and nuclei; **b**, naive integration of fetal data and uniform manifold approximation and projection (UMAP) showing **i**, clustering; **ii**, predicted doublets; **iii**, erythrocyte detection using a two-compartment Gaussian mixture model on the summed expression of erythrocyte genes *HBB, HBG1, HBG2, HBM, HBA2, HBA1, HBQ1, ALAS2* allowing to identify; **iv**, high erythrocyte fraction clusters for removal; validated by **v**, the summed expression of erythrocyte genes. **c**, Fetal samples contained a greater quantity of **i**, UMIs and; **ii**, unique genes expressed and were downsampled to 15 000 UMIs and; **iii**, the resulting relationship between depth and complexity was similar in both adult and fetal samples. Fetal datasets were integrated after erythrocyte removal as shown and clustered ready for subsampling and integration as shown in **d, i-v**, Louvain clustering using resolutions of 0.1–2 respectively. Resolution 0.5 was selected with 21 clusters, which when subsampled down to the number of epicardial cells identified using epicardial markers such as **vi**, UPK3B (n = 1598), produced approximately the same number of fetal cells as subsampled adult cells. Integration of subsampled adult and fetal data; **e**, was performed hierarchically by prioritising donors in a custom integration tree. Distributions for each box in 1c(i-ii) were drawn from n = 1562, 3504, 5074, 1479, 3454, 1866, 2845, 2124, 3400, 959, 3763, 4226, 2072, 1709, 1941, 1919, 8023, 724, 3630, 1832, 576, 8597, 5460, and 6764 cells respectively in the order of plotted groups. Each distribution’s centre horizontal line denotes population median, while box edges and whiskers are drawn at 1 and 1.5 × interquartile range respectively.

**Fig. 1:**
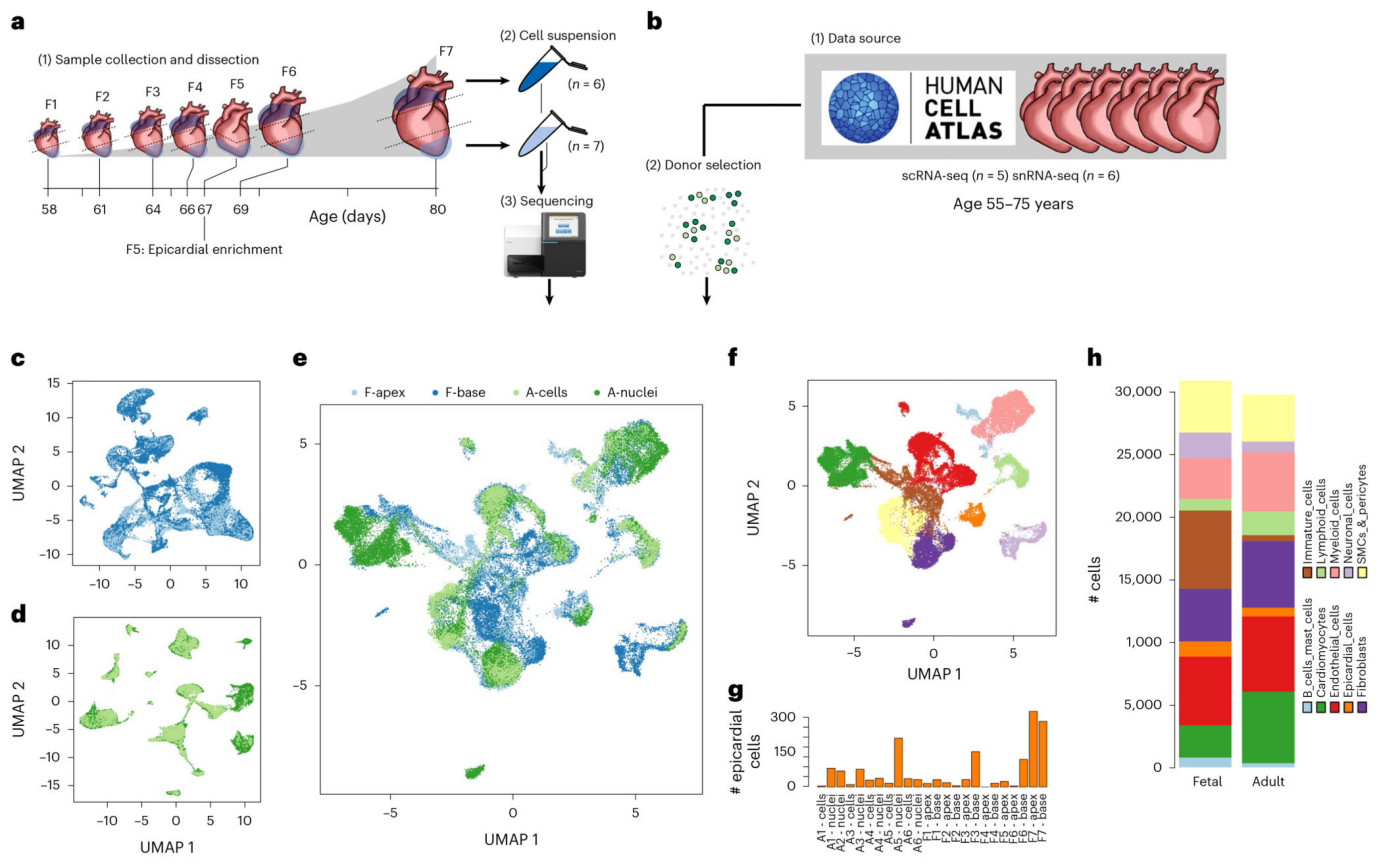
Composition and integration of adult and fetal hearts. Single-cell data were collected from sequencing of base and apex samples from seven fetal hearts (**a**) and publicly available data of six healthy adult hearts (**b**). UMAP embeddings of the remaining cells after quality control show integrated fetal apex with base (**c**) and integrated adult cells with nuclei (**d**). Stage integration and dimension reduction of all sources show the overlap of stage and source (**e**) and low-resolution clustering of cell types (**f**). This clustering shows the number of epicardial cells found across all samples (**g**) and the basic cell type composition of both sampled hearts used in this analysis (**h**).

Seven healthy fetal hearts between 8 weeks and 12 weeks post-conception were dissected, taking the base and apex from six donors and the apex attached to peeled epicardium from one (Fig. 1a). These 13 fetal samples were dissociated, sequenced and aligned using Illumina’s 10x scRNA-seq platform. We removed erythrocytes (Extended Data Fig. 1) and predicted doublets and lower-quality cells to obtain transcriptomes for 47,473 fetal cells (Supplementary Table 1). In parallel, scRNA-seq and single-nucleotide RNA sequencing (snRNA-seq) data from six healthy adult hearts containing 37,462 cells and 153,053 nuclei were obtained from the Heart Cell Atlas, selecting donors D2–D7 that contained at least three mesothelial annotated cells^22^ (Fig. 1b). We subsampled the datasets before downstream integration to equalize group sizes and reduce unwanted variability using multiple stratifications (Methods). This resulted in a more balanced distribution of cell types, donors and nuclei and amplified rarer cell type populations (Extended Data Fig. 1). After sampling, we were left with 29,779 transcriptomes from adult heart cells or nuclei and 30,889 transcriptomes from fetal heart cells (Supplementary Table 1).

We then integrated the samples hierarchically using a reciprocal principal component analysis (RPCA) integration pipeline from R package Seurat, combining sources within each donor first (Extended Data Fig. 1). We noted a reasonable degree of overlap between sources at this step (Fig. 1c,d). We then integrated the adult and fetal datasets, resulting in a correspondence between adult and fetal cells within clusters (Fig. 1e). However, fetal cells were more loosely distributed between the well-defined adult clusters, suggesting quantities of unspecified and immature states of cell types still progressing toward their mature adult equivalent (Fig. 1e). We began with low-resolution clustering using the Louvain method of community detection to label basic cell type annotations, arriving at ten low-resolution clusters (Fig. 1f), and used differential expression analysis and previous adult annotations to assign cell type labels and define the epicardial cell cluster (Fig. 1f). We noted that the number of epicardial cells was highly varied among donors, with most being found in fetal sample F7. However, there was no significant difference between the number of epicardial cells in fetal or adult donors, suggesting that the proportion of epicardial cells is not markedly different between stages (unpaired Student’s *t*-test; *P* > 0.05) (Fig. 1g). Overall, most clusters were distinct; however, one cluster appeared to bridge between multiple other cell types and expressed an ambiguous range of developmental markers. This cluster was composed mostly of fetal cells, which suggests that these were largely unspecified immature cells (Fig. 1h).

### Epicardial cells expressing EMT genes were absent in adult hearts

**Extended Data Fig. 2:**
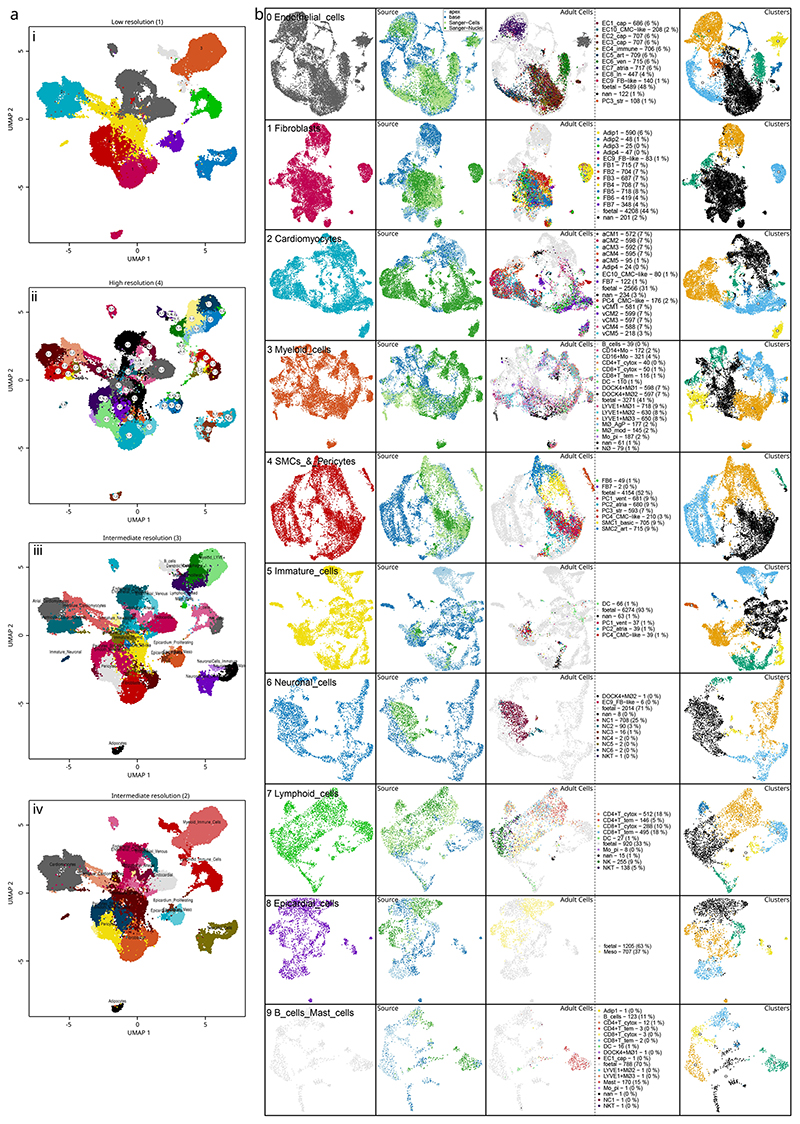
Annotation and clustering of integrated heart samples was carried out over several resolutions. Integrated adult and fetal data were iteratively clustered by aggregating high-resolution sub-clusters as shown in **a**, uniform manifold approximation and projection (UMAP) embeddings showing **i**, 10 initial low resolution clustering followed by; **ii**, sub-clustering, and **iii-iv**, aggregation of biologically similar cell clusters over two intermediate resolutions. Sub-clustering of low-resolution clusters depicted with; **b** subcluster UMAPs illustrating the identification of cell types and states using previous adult annotations, cell sources, and unsupervised clustering.

**Fig. 2:**
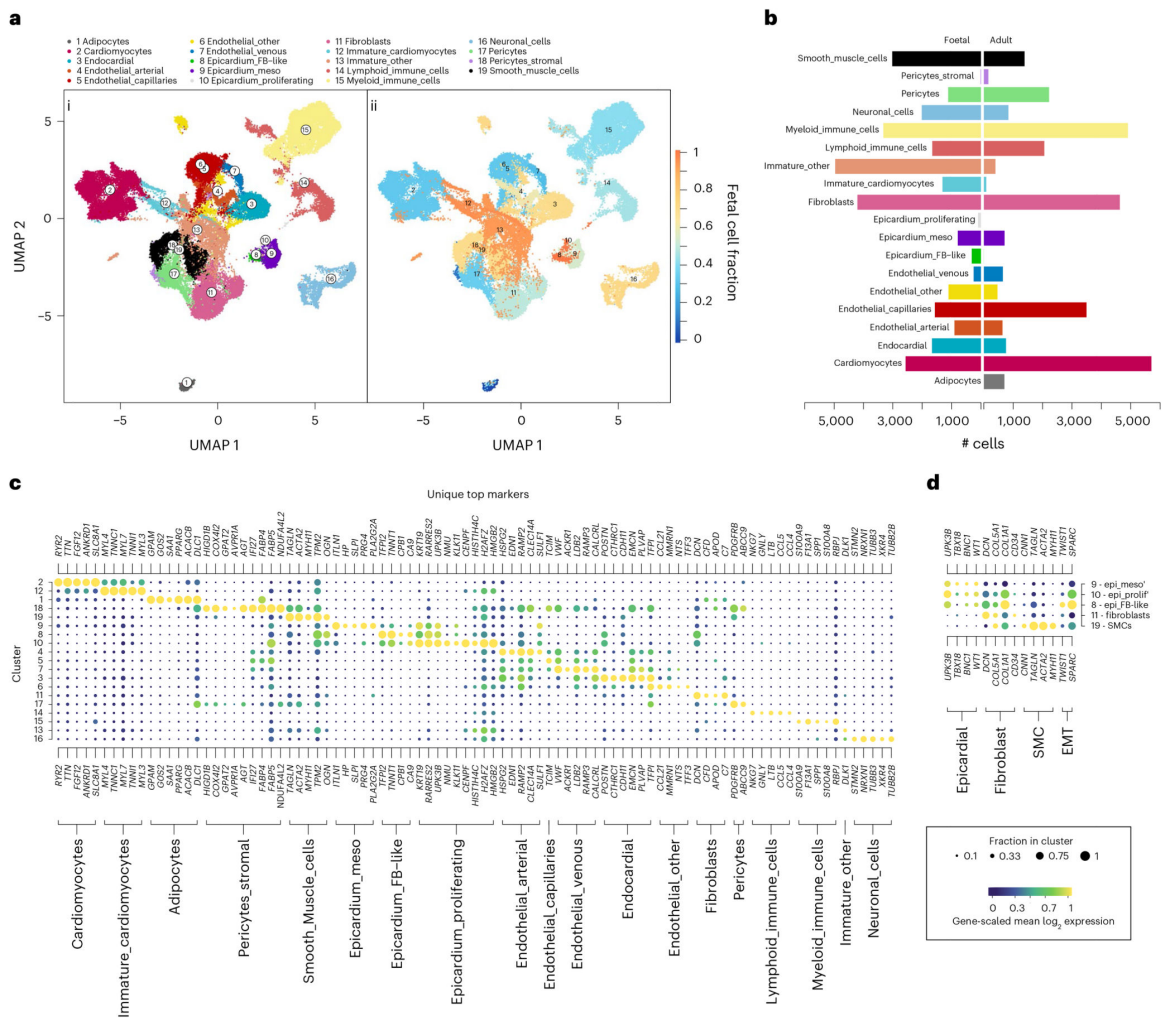
Identification of fetal-specific epicardial cells in human hearts. Higher-resolution clustering of the integrated dataset reveals distinct stage-specific epicardial and other cell type populations as shown in **ai**, a two-dimensional UMAP of cluster assignments, and the fraction of fetal cells within each cluster (**aii**). The absolute number of cells across fetal or adult conditions in **b**—a bidirectional bar chart shows stage bias of each cluster. Differential expression analysis between clusters with the top upregulated genes in **c** shows marker genes used in cluster annotation and identification. **d**, The expression of established markers of epicardial, fibroblast, smooth muscle cell (SMC) and EMT markers within epicardial subpopulations.

We performed subclustering and iteratively aggregated these sub-clusters together across several resolutions (Extended Data Fig. 2). We selected an intermediate resolution of 19 clusters for downstream analysis where the epicardium was divided into three subpopulations: 8, 9 and 10 (Fig. 2ai). Interestingly, we measured the fraction of fetal cells and found that epicardial clusters 8 and 10 comprised 0.6% and 0% adult cells, respectively, suggesting fetal specificity. However, cluster 9 was equally split between stages with 47.2% adult cells, suggesting an age-persistent epicardial cell type (Fig. 2aii,b). We carried out a differential expression analysis among all clusters and combined the upregulated markers in each cluster with previous annotation of adult cells to determine the cell types present across both ages (Fig. 2c, Extended Data Fig. 2 and Supplementary Table 2). In non-epicardial cells, we found that adipocyte cluster 1 and stromal pericyte cluster 18 were almost entirely adult cells (Fig. 2aii,b). The three epicardial subclusters expressed well-established epicardial signature genes *KRT19*, *RARRES2*, *UPK3B*, *WT1* and *BNC1* (Supplementary Tables 3 and 4). However, we labeled cluster 8 as Epicardium_FB-like in light of its expression of fibroblast genes, including *DCN*, *COL1A1* and *POSTN* (Fig. 2c,d); cluster 9 as Epicardium_Mesothelial after previous annotation and broad epicardial gene expression; and cluster 10 as Epicardium_Proliferating with its expression of cell cycle and mitotic genes *CENPF* and *HMGB2* (Fig. 2c). Interestingly, the Epicardium_FB-like cluster appeared to have low *TBX18* expression, unlike the other epicardial clusters, but expressed genes *TWIST1* and *SPARC*, which are strongly associated with EMT (Fig. 2d). Based on this evidence, we describe the fibroblast-like epicardial cells in cluster 8 as a transient population of mesenchymal EPDCs not yet differentiated into epicardium-derived lineages, which may not be present in the quiescent adult heart.

### Aging epicardium loses many fetal epicardial gene programs

**Extended Data Fig. 3:**
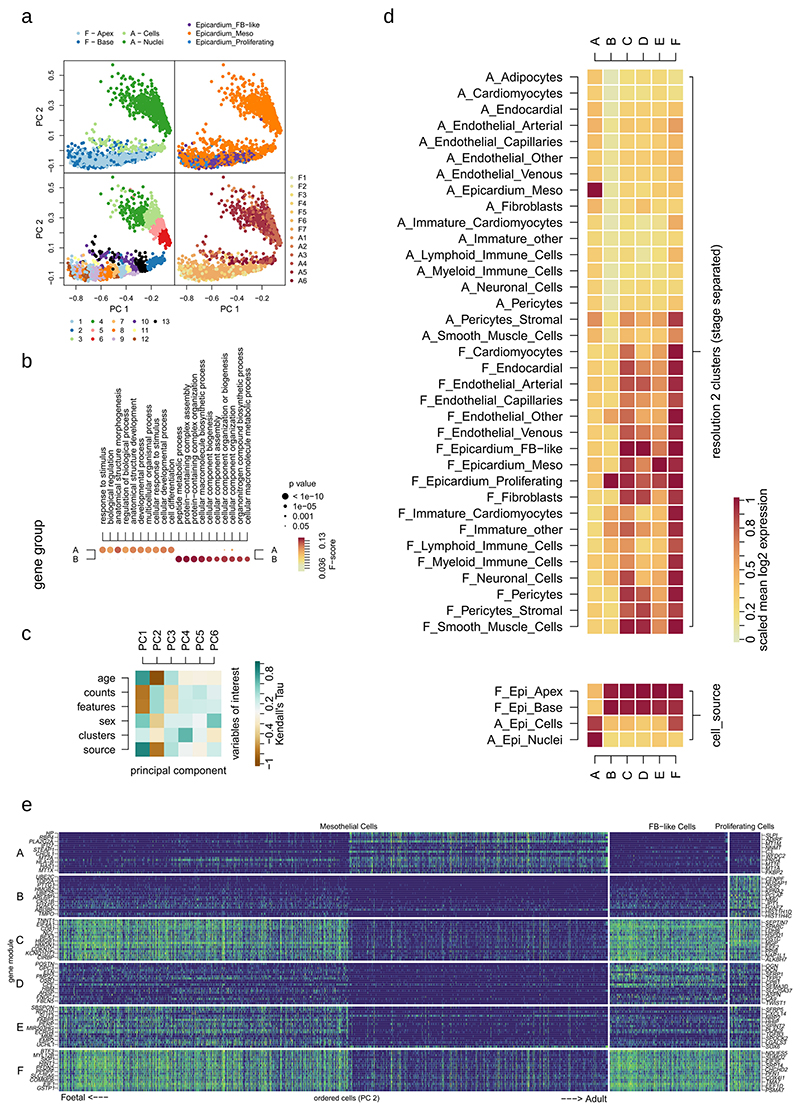
Further observations and technical hurdles in generating epicardial gene modules. The gene expression matrix of epicardial cells was isolated from the rest and binarised. Co-occurrence clustering of genes including nuclei in the matrix created two gene modules and a, principal component analysis (PCA) on cellular commitment to modules confirms that the main separation of the dataset was between cells and nuclei. Gene Ontology enrichment was carried out; b, revealing broad terms associated with nuclear or cytosolic compartments. Use of Kendall’s rank correlation; c, between cell variables and PCA components with components 2 and 4 highly correlating with age and clusters resepctively as variables of interest for visualisation. d, the mean expression of the 6 gene modules in the 19 resolution-2 clusters as well as cells grouped by source and; e, the expression of the top 20 genes in each gene module across age-ordered epicardial cells (age component PC2).

**Fig. 3:**
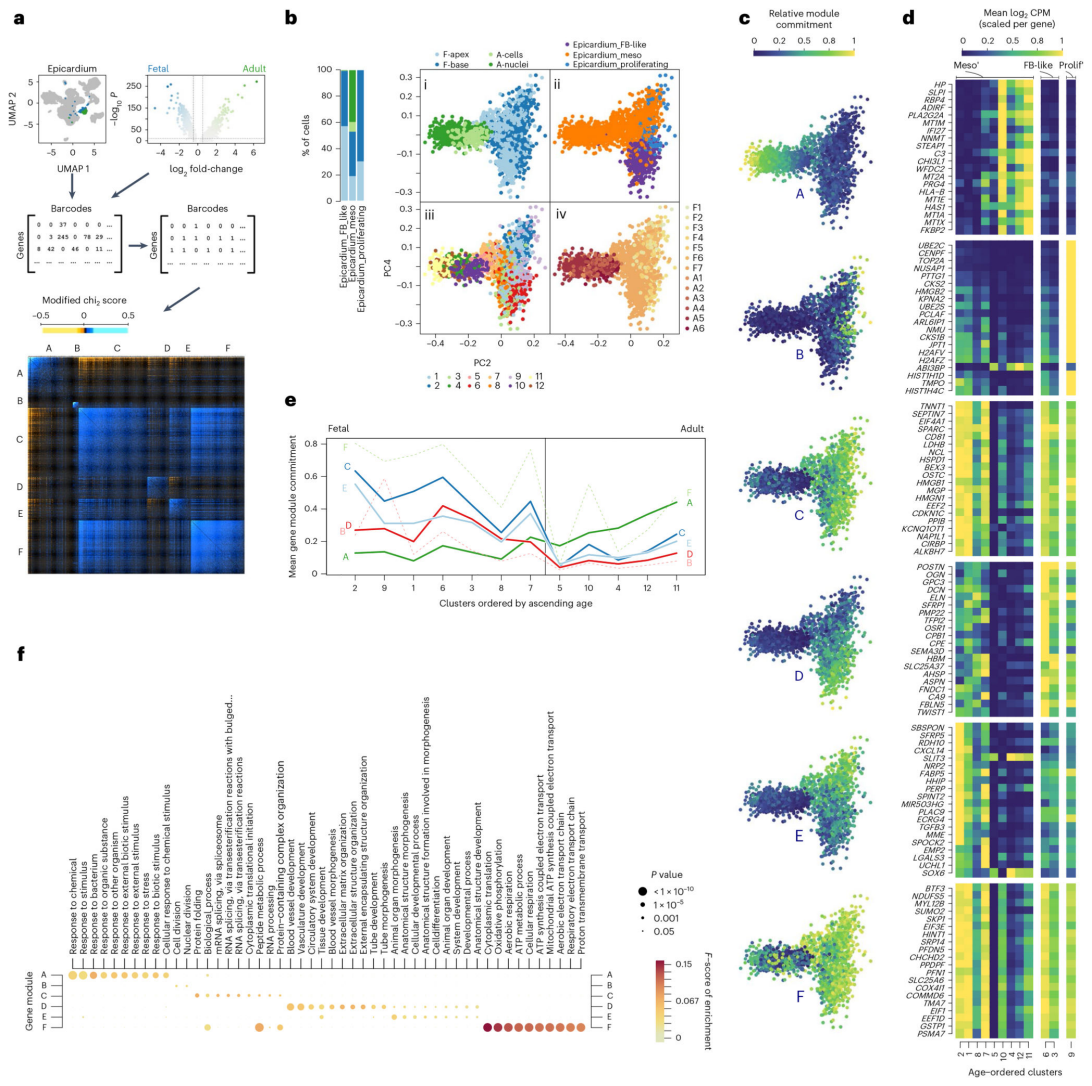
Different human epicardial states are determined by gene modules. **a**, Gene modules of the human epicardium were identified through adult versus fetal differential expression analysis and co-occurrence clustering. **b**, Epicardial cell states were identified using PCA, illustrating the cell sources (**bi**); previous clustering of the heart cells at resolution 2 (**bii**); clustering across gene module commitment (**biii**); and ranked age of samples (**biv**). Further PCA plots in **c** show the commitment of epicardial cells toward each gene module, and age-ordering of epicardial cells shows the age-associated changes in gene expression in the top 20 gene from each module (**d**) and the age-associated changes in commitment toward each module across epicardial clusters (**e**). **f**, Dot plot depicts Gene Ontology biological process term enrichment for each module, showing scores of significance and harmonic mean of recall and precision. Gene set significance was calculated using hypergeometric tests (background genes, *n* = 27,956) and adjusted for multiple comparisons using gprofiler’s g:SCS algorithm.

We isolated epicardial clusters 8, 9 and 10 and identified six distinct transcriptional modules of co-expressed genes by analyzing dropout patterns only in the cells (Fig. 3a). Cellular commitment toward each module was calculated as a new feature for principal component analysis (PCA) where age and cell type were orthogonally represented by components 2 and 4 (Fig. 3b); component 1 appeared to be unwanted technical variation (Methods, Extended Data Fig. 3). Finally, we re-clustered all epicardial cells into 12 states by their commitment to each gene module using the Louvain method of community detection (Fig. 3biii,c and Supplementary Table 5) and ordered them by mean cellular ranked-age to reveal changes in module commitment and genes caused by aging (Fig. 3d,e and Extended Data Fig. 3). We found only one aging-associated module (A) including the genes *HP* (haptoglobin), *SLPI* (secretory leukocyte peptidase inhibitor) and *PLA2G2A* (phospholipase A2 group IIA) (Fig. 3d,e). Commitment to module A was initially low in early fetal states at 13% and increased throughout development, peaking at 44% in adult cells (Fig. 3e). However, fetal epicardium was committed to many distinct modules (B to E). Module B was seen exclusively in the epicardial state overlapping with Epicardium_Proliferating cells (Fig. 3d,e), whereas all fetal epicardial cells appeared to be highly committed to module C, containing genes such as *TNNT1* (troponin T1, slow skeletal type), *SPARC* (secreted protein acidic and cysteine rich) and *MGP* (matrix gla protein). Adult cells expressed between only 4% and 25% of module C genes.

Our most interesting finding was in module D, with high commitment in Epicardium_FB-like cells belonging to epicardial states 6 and 3, with 42% and 33% of its genes expressed, respectively (Fig. 3b,c,e). In contrast, adult mesothelial cells expressed between only 4% and 12% of the genes in this module. Module D contained fibroblast genes *POSTN* and *DCN* (Fig. 3d) and established markers of EMT, such as *TWIST1*, suggesting a signature of EPDCs. Commitment toward module E was seen mostly in fetal Epicardium_Mesothelial cells, including the genes *SBSPON* (somatomedin B and thrombospondin type 1 domain containing), *CXCL14* (C-X-C motif chemokine ligand 14) and *SFRP5* (secreted frizzled related protein 5). Lastly, module F may have been associated with technical variables, with more commitment seen in cells when compared with nuclei (Extended Data Fig. 3).

### Aging shifts epicardial focus from angiogenesis to immune response

We then examined the function of each gene module using GprofileR in an over-representation analysis across Gene Ontology biological processes. First, gene module D was enriched for pro-regenerative processes related to angiogenesis, EMT and wound repair, including blood vessel development, circulatory system development, angiogenesis, cell migration and extracellular matrix organization (Fig. 3f and Supplementary Table 6). Commitment of epicardium to module D suggests that the fetal epicardium is poised for angiogenic response, whereas the adult epicardium is not. Second, we noticed that module A was broadly enriched for processes involved in response to external stimuli, including response to stress, defense and immune response (Fig. 3f and Supplementary Table 6), suggesting that the human epicardium transitions toward an immune-responsive state with age. This unexpected result reveals an unexplored characteristic of epicardial aging, which may be important for cardiovascular regeneration. Lastly, we found broad developmental terms, including animal organ morphogenesis and cell differentiation, enriched in the early mesothelial module E and less-specific protein processing terms in fetal module C (Fig. 3f). Processes found in module B also validated our labeling of proliferative epicardium with the terms ‘cell division’ and ‘nuclear division’. In summary, these results suggest that aging reduces epicardial commitment toward regenerative angiogenic programs. In particular, it is important to note that the epicardial population most committed to the regenerative module D was absent in adults.

### Epicardial markers reveal WNT signaling in fetal epicardium

**Extended Data Fig. 4:**
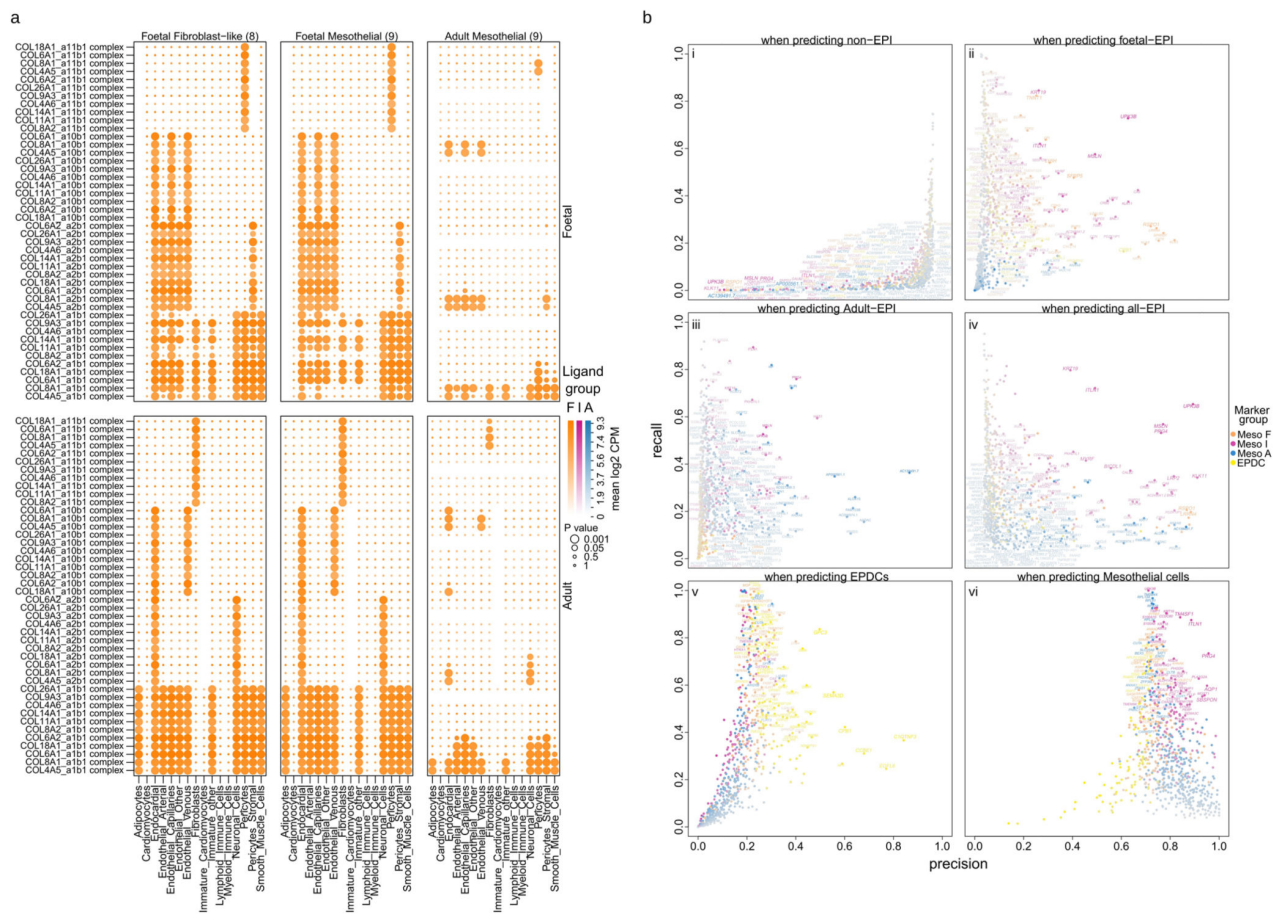
Epicardial-specific genes included many collagens forming a large component of epicardial-specific communication. Predictive ligand receptor analysis with CellPhoneDB of communication shown in a, from epicardial cells with other heart cell types with a large component of fetal-specific and epicardial selective collagens interacting both with fetal and with adult hearts. Significance of each interaction was calculated in CellPhoneDB using the fraction of mean expression between gene pairs that are equivalent or higher than the gene-pair expression in 1000 random permutations. Recall and precision statistics for the positive expression of each epicardial marker was scored b.i-b.iv, on their abilities to predict non-epicardial cells, fetal, adult, or all epicardial cells, which was repeated; b.v & b.vi, in only UPK3B positive cells in predicting either EPDCs or Mesothelial epicardial cells.

**Fig. 4:**
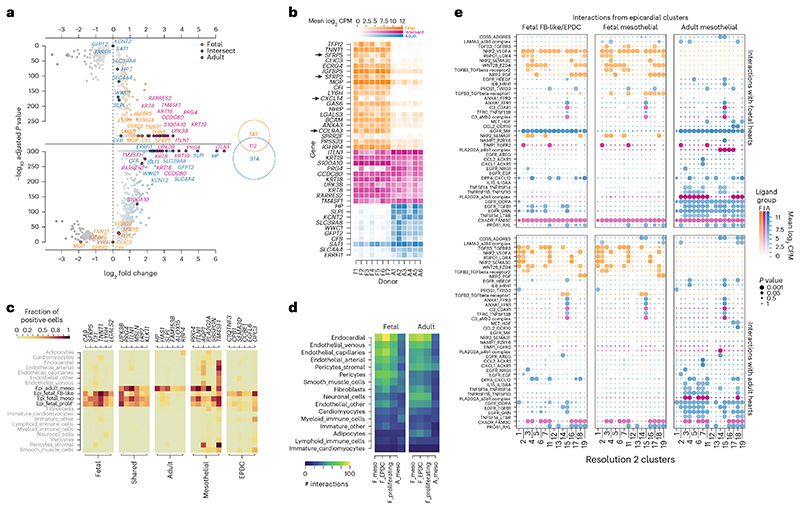
Communication specifically from the epicardium changes with age. Specific epicardial markers were identified as selective for fetal, shared or adult epicardium through a parallel and stage-independent analysis shown in a mirrored volcano plot highlighting the top 10 genes in each subset (**a**) and a heat map of expression of the top 20, 10 and 10 gene subsets where markers with clear fetal bias, including WNT signaling genes, are shown with arrows (**b**). **c**, Top-scoring predictive markers for epicardium compared with other heart clusters and between epicardial subtypes. The predicted level of epicardial communication with other heart cell types in each stage was calculated using CellPhoneDB, showing the total volume of communication (**d**) and clustered epicardial-specific secretions filtered by upregulated genes predicted to interact with all other heart clusters of either fetal or adult source by tissue, color-coded by stage selectivity of secreted markers (**e**). In **e**, x-axis labels refer to resolution 2 cluster IDs: 1, Adipocytes; 2, Cardiomyocytes; 3, Endocardial; 4, Endothelial_ Arterial; 5, Endothelial_Capillaries; 6, Endothelial_Other; 7, Endothelial_Venous; 11, Fibroblasts; 12, Immature_Cardiomyocytes; 13, Immature_other; 14, Lymphoid_Immune_Cells; 15, Myeloid_Immune_Cells; 16, Neuronal_Cells; 17, Pericytes; 18, Pericytes_Stromal; 19, Smooth_Muscle_Cells. The significance of epicardial markers in **a** was determined using two-sided Wilcoxon rank-sum tests and adjusted for multiple comparisons using Bonferroni correction.

We then evaluated the age selectivity of epicardial-specific genes by carrying out differential expression analyses within each stage. After removing genes describing non-epicardial differences between adult and fetal stages, we found 633 genes upregulated in the epicardial cluster when compared with other heart cell clusters (Wilcoxon rank-sum; *P*<1×10^−10^, log_2_ fold change > 0.5), constituting 147 fetal markers, 374 adult markers and 112 markers of both stages (Fig. 4a,b and Supplementary Table 7). We also ranked the epicardial genes by their ability to predict epicardial cells using precision, recall and *F*-score (Fig. 4c, Extended Data Fig. 4 and Supplementary Table 8).

First, we found several genes markedly absent in adult epicardium: *SFRP5* (secreted frizzled related protein 5), *SFRP2*, *CXCL14* and *COL9A3* (collagen type IX alpha 3 chain) (Fig. 4b). Second, the fetal epicardium was best predicted by the shared marker *CA9* (carbonic anhydrase 9) (*F*-score = 0.51, precision = 0.3, recall = 0.81), followed by *SFRP5, CFI* (complement factor I), *TNNT1*, *LY6H* (lymphocyte antigen 6 family member H) and *LGALS2* (galectin 2). Most interestingly, we found that *SFRP5* was one of 11 other fetal genes within the Gene Ontology process canonical Wnt signaling pathway, including *SFRP2*, *WNT2B* (Wnt family member 2B), *RSPO1* (respondin-1) and *FGF9* (fibroblast growth factor 9). *SFRP2* and *SFRP5* are soluble pleiotropic modulators of WNT signaling and may be essential in myocardial repair^25,26^, whereas *WNT2B* is a canonical WNT ligand found to increase zebrafish cardiomyocyte proliferation after injury^27^. Additionally, *RSPO1* was recently implicated in cardiomyocyte compaction during development and identified in the epicardium of regenerating P1 neonatal mice but not in non-regenerating P7 mice^12,28^. Lastly, *FGF9* is implicated in epicardial-mediated regenerative signaling, including vasculogenesis^29^. Functionally, this WNT component is of utmost relevance to epicardial-mediated regeneration. Other notable fetal markers may promote regeneration, such as *CA9* stimulating cell migration under hypoxia in mice^30^ as well as *BMP3* (bone morphogenetic protein 3) and *TGFB3* (transforming growth factor beta 3).

Irrespective of age, our library identified *UPK3B* (uroplakin 3B) as the most selective epicardial marker validated by reports of its robust expression^23,31,32^ (Fig. 4c). This was followed by two established markers of epicardium: *ITLN1* (intelectin-1) and *MSLN* (mesothelin). Interestingly, *ITLN1* was in adult module A, linking our results with a clinically observed correlation of serum omentin-1 with age^33^. We also identified markers of the epicardium, such as *KLK11* (kallikrein related peptidase 11), *CALB2* (calbindin 2) and *SMPD3* (sphingomyelin phosphodiesterase 3) (Supplementary Table 8). In the adult epicardium, we found that *HP* was the best predictive coding gene (*F*-score = 0.44, precision = 0.3, recall = 0.81) (Fig. 4c), followed by *HAS1* (hyaluronan synthase 1), *SLPI* (secretory leukocyte peptidase inhibitor), *FAM153B* (family with sequence similarity 153 member B), *ALOX15* (arachidonate 15-lipoxygenase) and *RBP4* (retinol-binding protein 4). *HP* was seen in older human fetal epicardium^34^, suggesting a marker of maturing mesothelial cells that persists into adulthood (Fig. 4c).

Lastly, we found no clear marker of the fibroblast-like epicardial population. However, we used *UPK3B* as a pan-epicardial marker and repeated scoring between fibroblast-like cluster 8 and mesothelial cluster 9 using only *UPK3B^+^* cells (Extended Data Fig. 4 and Supplementary Table 8). We found that mesothelial-specific *PRG4* (proteoglycan 4) and *ITLN1* suggest spatial separation of epicardial cell types as *PRG4* encodes lubricin secreted into pericardial fluid^35^, and *ITLN1* encodes omentin-1 associated with epicardial adipose tissue (Fig. 4c). Without these proteins, EPDCs may be deeper within the myocardium than their *PRG4*-producing counterparts. Other mesothelial genes were *AQP1*, *PLA2G2A*, *SBSPON* and *TM4SF1* (Fig. 4c). In the EPDCs, we found *SEMA3D*, involved in concerted endothelial cell migration^36^; *CCBE1*, important in mouse cardiac development^37^; *EGFL6*, associated with angiogenesis^38^, and *GPC3*, reported to modulate WNT signaling^39^. Overall, these results suggested further that these epicardial cells are EPDCs no longer on the surface of the heart. Additionally, we showed that, although aged epicardium maintains a recognizable identity, it departs from our previous understanding and that aging establishes a novel epicardial state. Lastly, these results continued to show that key regenerative signaling is absent in adult epicardium, including angiogenic WNT signaling.

### Adult hearts still respond to fetal epicardial signaling

To examine the effects of restoring fetal states to adult epicardium, we predicted paracrine interactions from the epicardium using Cell-PhoneDB. First, we found more communication from fetal epicardium in comparison with adult epicardium when interacting with other cells of the adult heart. By restoring fetal states, the largest predicted increase in epicardial communication was seen in endocardial, venous endothelial and neuronal cell populations given by 88, 66 and 62 interactions, respectively, from fetal Epicardium_FB-like compared with 30, 27 and 16 interactions from adult Epicardium_Mesothelial (Fig. 4d). We then filtered these interactions for secreted epicardial-specific proteins from the differential expression analysis (Fig. 4a,b) and found that the volume of predicted communication between fetal epicardial and adult endocardial cells could be pro-angiogenic (Fig. 4e). Our results show that this was an age-associated loss of epicardial secretions as opposed to a loss of receptivity by adult hearts, as low communication from adult epicardium persisted even when interacting with the fetal heart, whereas both adult and fetal hearts received similar signals from fetal epicardium (Fig. 4e). Specifically, signaling from fetal epicardium consisted of *NRP2*-mediated signaling with endothelial *VEGF*s. However, it is unknown whether epicardial *NRP2* is soluble or membrane-anchored as part of this established pathway of angiogenesis^40,41^. We also found further evidence of WNT signaling from the epicardium, with *WNT2B* communication between fetal epicardium and *FZD4* found on adult endocardial, endothelial and stromal pericyte clusters. Reduced *FZD4* activity has been seen to markedly decrease vascular density in kidneys^42^. These interactions corroborate fetal angiogenic potential while highlighting target cells for epicardial WNT signaling. To our surprise, only a small volume of communication was seen with cardiomyocytes from both adult and fetal epicardium (Fig. 4d). One of these was another WNT signaling protein, *RSPO1*, predicted to interact with *LRG4* in cardiomyocytes as well as smooth muscle cells, adipocytes, fibroblasts and neuronal cells (Fig. 4e). Lastly, we found *TGFB3* signaling in fetal but not adult epicardium, agreeing with previous observations of low expression in adults^43,44^. Of relevance to angiogenesis, *TGFB3* was decreased in a low-EMT model of mouse epicardium while correlating with reduced vascular density of adjacent myocardium^32^, and its elevated expression after MI might reduce scarring after injury^45^.

Interestingly, many fetal interactions were from epicardial-specific collagens, such as *COL11A1* and *COL9A3* (Extended Data Fig. 4), which may play an important part in epicardial-mediated matrix reorganization. Another epicardial collagen, *COL3A1*, was also found here but was upregulated more broadly in fetal hearts when compared with adults and was omitted (Supplementary Table 7). Lastly, the adult epicardium was predicted to communicate with adult endothelial cells via *EGFR*, a complex and pleiotropic regulator of proliferation and survival of myocardial tissue (Fig. 4e). However, we could not determine if epicardial *EGFR* was a soluble form. Other adult epicardial interactions agree with adult immuno-inflammatory focus, with members of the TNF ligand or receptor superfamilies *TNFSF14* or *TNFRSF11B*, interleukins *IL15* and *IL6* and chemokines *CCL2* and *CXCL1* seen to interact with receptors on endothelial cells as well as other cells of the adult or fetal heart. Additionally, adult epicardial *PLA2G2A* interacting here with integrin complexes has previously been associated with coronary heart disease and infarction^46^. These results provide evidence that an in situ reactivation of fetal epicardial programs might increase regenerative communication with endothelial cells to drive angiogenesis and vascularization, which are key processes in cardiac regeneration.

### hESC-derived epicardium closely resembles fetal epicardium

**Extended Data Fig. 5:**
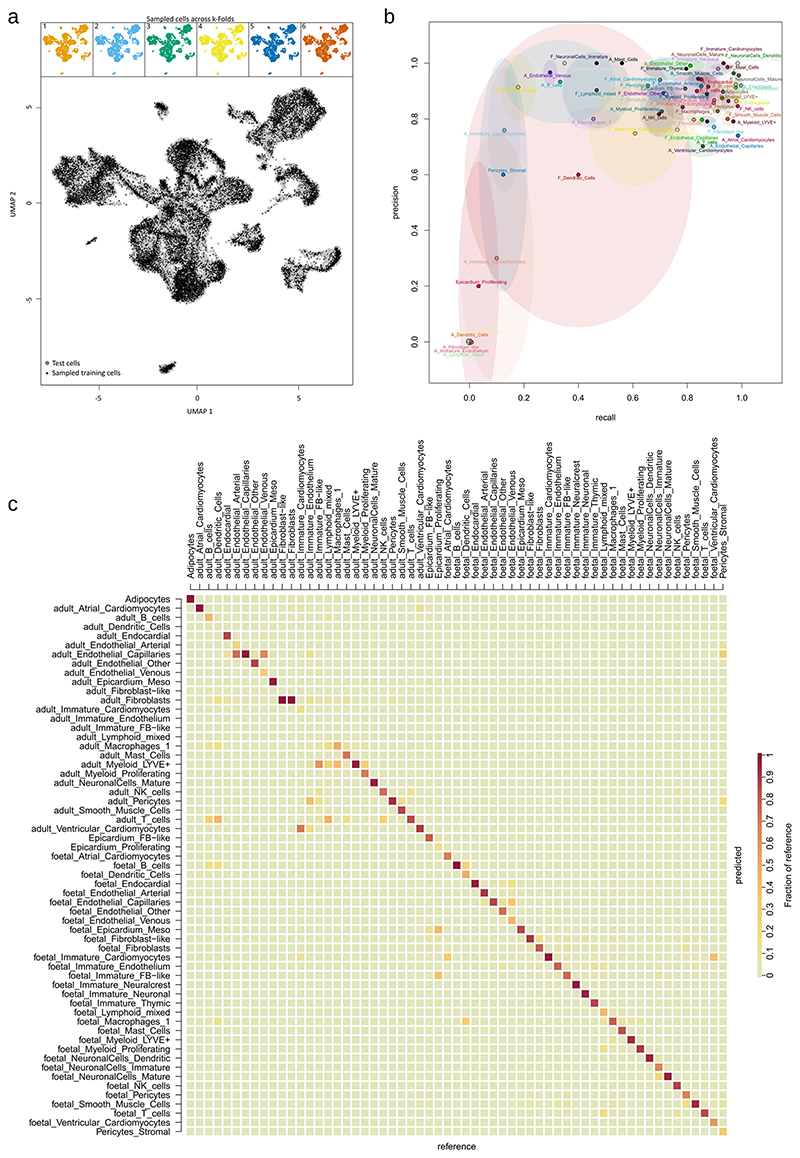
Training and validation of a random forest on high-resolution cell types. A training dataset cells for random forest classification was sampled as shown by a, the uniform manifold approximation and projection (UMAP) of integrated data. Sampled cells were shown in black covering 33 % of the dataset and with 6 folds representing the dataset evenly as shown by the coloured sub-plots. Cross-validation (6-folds) suggests reasonable model accuracy for most clusters by comparing b, precision and recall (mean and standard deviation, k = 6). Prediction of the remaining naive 66 % of cells as test data shown in c, the confusion matrix between the reference and predicted cell classes with high scores for most cell types.

**Fig. 5:**
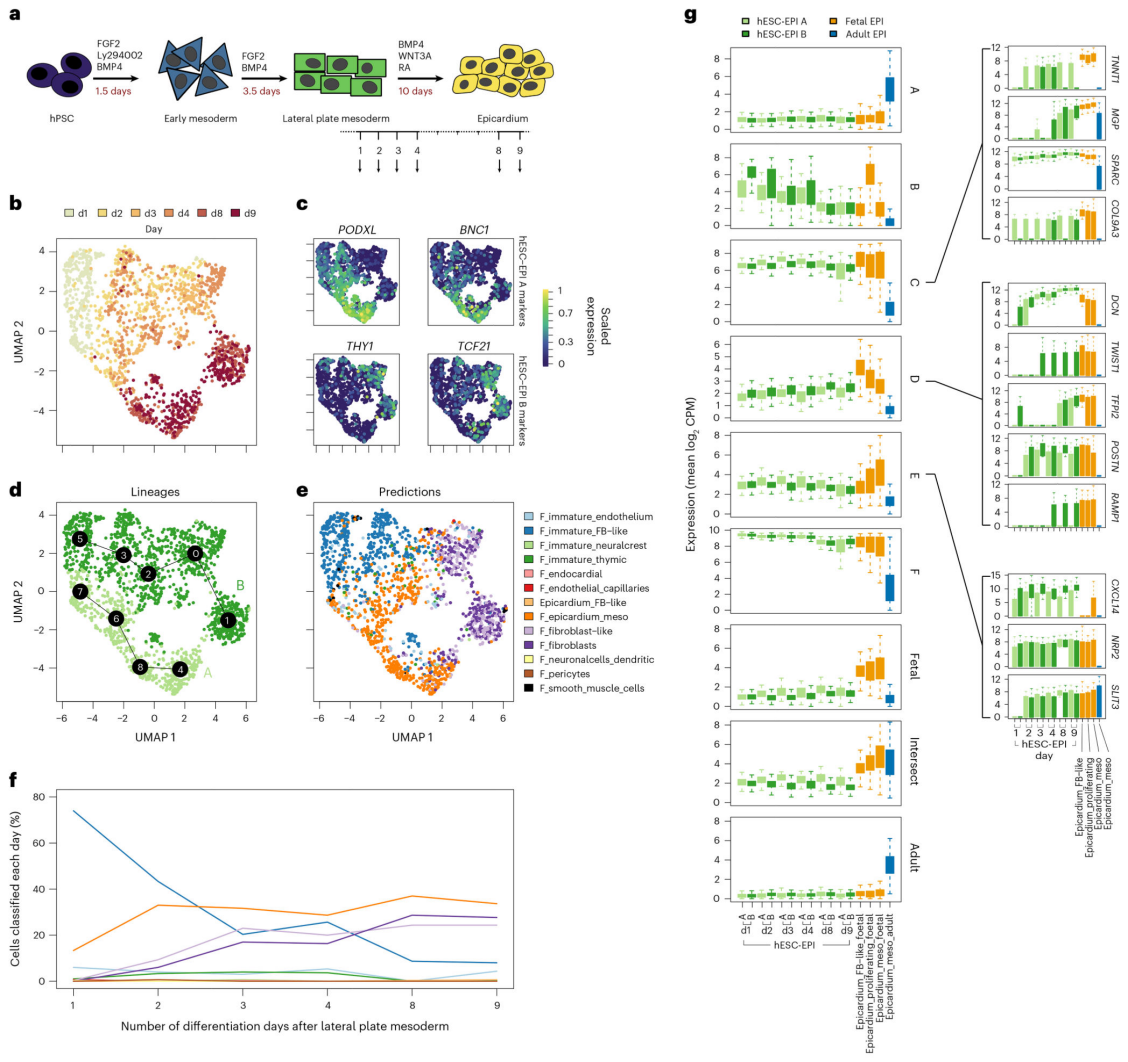
Stem-cell-derived epicardium models the in vivo epicardium. **a,** The last 9 days of an hESC-EPI differentiation were sampled during scRNA-seq. UMAP shows differentiation day (**b**); expression of previously identified sources of epicardial heterogeneity (**c**); lineage separation (**d**); and random forest classification of hESC-EPIs using an in vivo trained model (**e**). Differentiation population dynamics are shown in **f**, over the course of differentiation. **g**, hESC-EPI has a similar gene expression to fetal epicardium, the expression of top 50 markers from the six in vivo epicardial gene modules A to F and epicardial-specific genes from fetal, shared or adult sets. Within these groups, notable epicardial genes *TNNT1*, *MGP*, *SPARC*, *COL9A3*, *DCN*, *TWIST1*, *TFPI2*, *POSTN*, *RAMP1*, *CXCL14*, *NRP2* and *SLIT3* are highlighted. Each distribution’s center horizontal line denotes population median, and box edges and whiskers are drawn at 1 and 1.5× interquartile range, respectively. Distributions for each box in **g** were drawn from *n* = 94, 206, 73, 227, 56, 244, 45, 255, 137, 163, 165, 135, 315, 100, 790 and 705 cells, respectively, in order of plotted groups.

We previously harnessed active epicardium to augment heart regeneration using hESC-EPIs in situ^17^. However, the mechanisms governing this therapeutic success were unknown. To address this and identify commonalities between in vitro and in vivo epicardium, we harvested hESC-EPIs during the final 9 days of differentiation^47^ and generated an scRNA-seq time course (Fig. 5a,b). This protocol yields a heterogeneous epicardium^21^, confirmed in our results as a divergent differentiation into two branches, which expressed either *PODXL* and *BNC1* (lineage A) or *TCF21* and *THY1* (lineage B) (Fig. 5c,d). To determine how well hESC-EPI models in vivo epicardium, we trained a random forest classifier on the adult and fetal in vivo heart scRNA-seq high-resolution clusters and found that the number of epicardial predictions increased over differentiation, occurring in lineage A (Fig. 5e,f). In contrast, lineage B became classified as fibroblasts or fibroblast-like cells (Fig. 5e,f). Performance of our model was assessed using six-fold cross-validation where the non-proliferating epicardial clusters were predicted with high accuracy (Extended Data Fig. 5). These results reflect the separation of Epicardium_Mesothelial and Epicardium_FB-like populations found in vivo. Both populations appeared to stem from a population predicted as fetal Immature_FB-like cells (Fig. 5f).

We then calculated the mean expression of epicardial gene modules during the hESC-EPI differentiation and found that angiogenic module D increased throughout, reaching a similar expression to the in vivo fetal epicardium (Fig. 5g). In contrast, adult module A was absent in hESC-EPIs (Fig. 5g). We also noticed that the expression of the more mesothelial gene module E appeared to be higher in the *PODXL*^+^ branch A, agreeing with random forest predictions. We also observed a transiently high initial but decreasing expression of the proliferation-associated gene module B (Fig. 5g). Additionally, we found that the expression of many epicardial-specific genes increased throughout differentiation, including *TNNT1*, *MGP*, *SPARC* and *COL9A3* in module C; *DCN*, *TWIST1*, *TFPI2*, *POSTN* and *RAMP1* in module D; and *CXCL14*, *NRP2* and *SLIT3* in module E (Fig. 5g). Lastly, the in vitro model also expressed several epicardial WNT signaling genes, such as *SFRP2*, *WNT2B* and *TFPI2*, seen more obviously in higher-depth sequencing data of hESC-EPIs produced following the same protocol^21^. Conservation of these genes across both in vivo and in vitro systems implies similar epicardial function in both environments and highlights in vivo pathways that hESC-EPIs may use when augmenting hESC-cardiomyocyte grafts^17^.

### EPDCs are found within the myocardium and sub-epicardial space

**Extended Data Fig. 6:**
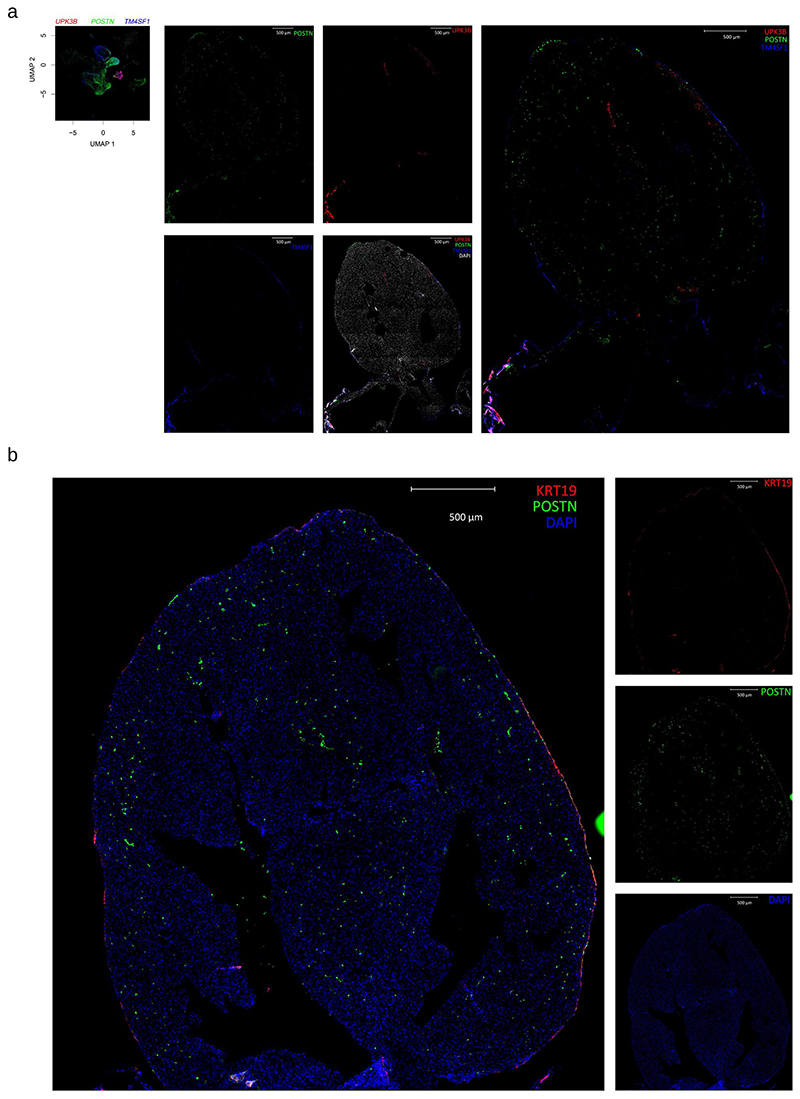
Predicted separation between EPDCs and Mesothelial epicardium with UPK3B, TM4SF1, and POSTN using a, pseudostain on integrated uniform manifold approximation and projection embeddings followed by immunocytochemical staining using antibodies. The UPK3B antibody did not label the epicardium clearly as a result of undefined technical difficulties. Lower-magnification imaging of b, immunofluorescence using antibodies for KRT19 and POSTN as one strategy for distinguishing between EPDCs and Mesothelial epicardium in fetal cardiac tissue. Images shown are representative of stains carried out on three sections with each combination on a single heart (a, BRC2281; b, BRC2375).

**Extended Data Fig. 7:**
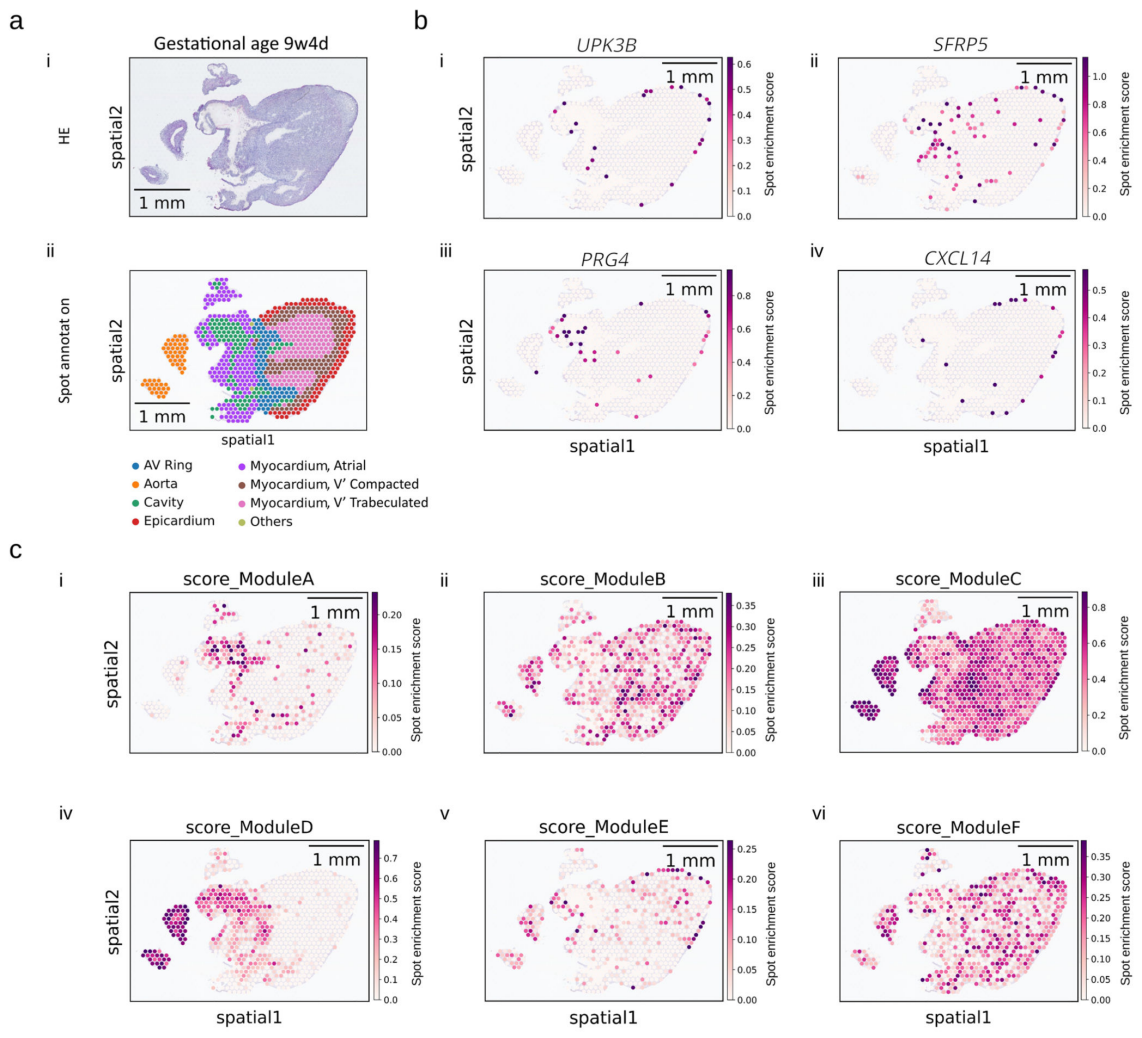
Spatial transcriptomics of a fetal heart aged 9 week 4 days post-conception showing; a, H&E staining and spot annotation, bi-iv, epicardial markers; and ci-vi, expression scores of epicardial gene modules as calculated from the top 20 genes in each module. This experiment was carried out once.

**Fig. 6:**
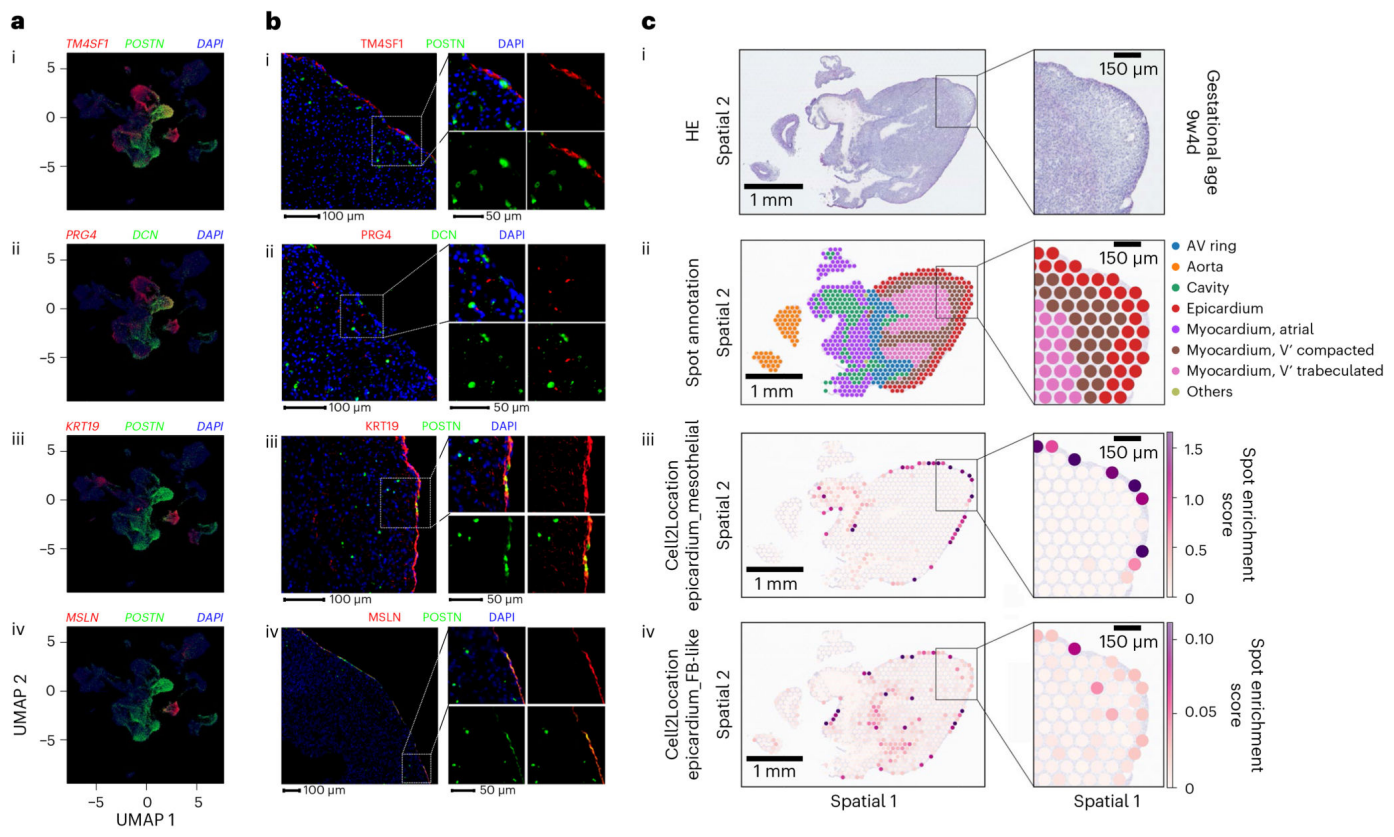
EPDCs were found in sub-epicardium and myocardium. **a**, Spatial validation of this cluster as EPDCs was carried out using a pseudostain targeting strategy from UMAP of scRNA-seq data, with gene expression displayed in RGB color space and a constant blue channel value of 0.2 for heuristic DAPI simulation. **b**, Staining of fetal hearts using double stains for *TM4SF1* and *POSTN* (**bi**), *PRG4* and *DCN* (**bii**), *KRT19* and *POSTN* (**biii**) and *MSLN* and *POSTN* (**biv**). Images shown are representative of stains carried out on three sections, with each combination on a single heart: BRC2281 (**bi** and **bii**) and BRC2375 (**biii** and **biv**). **c**, Epicardial clusters were mapped onto spatial transcriptomics data from a single fetal heart using the Cell2Location algorithm. 9w4d, 9 weeks, 4 days.

Lastly, we used immunocytochemistry to spatially resolve fetal epicardial cell populations in a strategy combining new markers from our analysis and *POSTN* (periostin) highly expressed in gene module D (Fig. 3d). We performed a uniform manifold approximation and projection (UMAP) pseudostain on the integrated dataset (Fig. 2a) for visualizing combinations of top epicardial markers in the red and green RGB channels (Fig. 6a) and selected *TM4SF1* and *PRG4* for separating Epicardium_FB-like and Epicardium_Mesothelial clusters; *POSTN* and *DCN* for identifying EPDCs; and *KRT19* and *MSLN* for the positive selection of EPDCs against fibroblasts or endocardial cells. Our *UPK3B* stain was ineffective (Extended Data Fig. 6). No co-localization was observed between *TM4SF1* or *PRG4* and *POSTN* or *DCN*, validating our pseudostain and scRNA-seq analysis (Fig. 6bi,bii). However, co-localization of *POSTN* was found with *KRT19* or *MSLN* on the epicardium, agreeing with shared fibroblast and epicardial genes seen in the scRNA-seq data (Fig. 6biii,biv). These double-positive cells were found in the mesothelial layer but remained negative for *PRG4* or *TM4SF1* and may be switching state and preparing for EMT (Fig. 6b). Furthermore, we found that *POSTN*^+^ cells were also sparsely distributed within the myocardial tissues and sub-epicardial layer, which indicates EPDCs that have lost mesothelial hallmarks. We also analyzed spatial transcriptomics in one fetal heart aged 9 weeks, 4 days and projected epicardial populations spatially using Cell2Location (Fig. 6c). We validated epicardial spots using markers found in this study (Extended Data Fig. 7) and also monitored the spatial distribution of epicardial gene modules. However, this was not informative, as modules were generated by comparing between epicardial states, not between epicardial cells and other cells of the human heart (Extended Data Fig. 7). We found that epicardial clusters were identified on the periphery of the myocardium as expected (Fig. 6c). However, although the Epicardium_Mesothelial cluster remained in spots on the surface of the heart, the Epicardium_FB-like cluster was enriched in spots deeper within the myocardium (Fig. 6c). Overall, these results provide further spatial evidence that our fibroblast-like epicardium is a transient migratory EPDC population that loses mesothelial identity after EMT, forming a key part of the developmental and regenerative dynamics absent in adult hearts.

## Discussion

Epicardial activity appears to be an important element of heart regeneration. On the one hand, active epicardium plays a substantial role in successful cardiac regeneration in adult zebrafish, newts and developing mammalian systems. On the other hand, the epicardium is reportedly quiescent in adult mammalian and human hearts, which lack regenerative capabilities. However, despite the apparent importance of the epicardium, few studies have yet defined how aging alters the regenerative programs in human epicardial cells, presenting an opportunity for finding novel therapeutic mechanisms in treating ischemic injury. In addressing this unexplored space, we combined and compared fetal and adult hearts from humans at single-cell resolution, to our knowledge for the first time, and focused on epicardial cells within them. We revealed both compositional and molecular differences between the adult and fetal epicardium that, in part, may underpin the limited regeneration seen in adult human hearts. We found that the adult epicardium (1) has a limited population of mesenchymal EPDCs; (2) has reduced paracrine communication; (3) lacks fetal-specific regenerative and angiogenic epicardial gene programs; and (4) is more primed for response to immune stimuli.

This is the first time that human EPDC transcriptomes have been described at a single-cell resolution, as determined by their expression of mesenchymal genes *TWIST1* and *SPARC* as well as the combined expression of known epicardial and fibroblast genes. These characteristics are shared with cell populations found in developing mouse and chick hearts^19,32^. Additionally, the position of these cells was consistent with migrating EPDCs using immunohistochemistry using antibodies for *POSTN*, *TM4SF1* and established epicardial markers. A major finding of our study was the lack of EPDCs in adult human cardiac tissue. This might be explained by quiescent adult epicardial cells being less responsive to EMT-driving stimuli as previously demonstrated in cultured adult EPDCs^48^. Current knowledge suggests that epicardial cells undergo EMT, forming EPDCs, which subsequently differentiate into other cardiac cell types^3,4^, resulting in a departure from epicardial cell states. Therefore, we hypothesize that fewer migrating adult epicardial cells results in a reduced quantity of transient EPDCs at steady state due to differentiation or efflux from the transient EPDC cell type.

Our results agree with current understanding of adult epicardial quiescence, and, although experiments have shown that adult epicardial cells may be pro-regenerative when transplanted, these cells are either primed or likely primed during culture conditions^14,49^. Our study is the first exhaustive documentation of the age-associated loss of epicardial signals involved in angiogenesis, proliferation and survival in healthy non-primed adult epicardium. For human heart regeneration, one strategy is to restore epicardial activity by reverting the adult epicardium to fetal states or by administering active epicardial cells generated from pluripotent stem cells^47^. Our study provides a roadmap for this translational effort, as, for the first time, we now have a transcriptome-wide description of the ingredients required to bring fetal-like regenerative function back into adult epicardium. First, the regenerative human epicardium may drive angiogenesis through *NRP2*, *VEGFA*, *CXCL14* and *SLIT3* with adult endothelial cells; new vessel growth is likely sourced from pre-existing endothelial cells^50,51^. Angiogenesis resulting from these interactions has been confirmed in mice where epicardial SLIT2-mediated co-localization with *ROBO4*-expressing endothelial cells was essential for vascularization^32^ and may also be important for angiogenesis in human tissue^51^. Furthermore, *SLIT*/*ROBO* signaling may also involve epicardial *CXCL12*/*CXCR4* (refs. 52,53) in an interaction that may include the early epicardial-specific gene and allosteric *CXCR4* and *CXCL14*. Interestingly, *CXCL14* has not been found in animal epicardium to date and may be a key difference between animal and human epicardial signaling. Second, we should also aim to reactivate paracrine WNT signaling, including *SFRP*s *2* and *5*, *RSPO1* and *WNT2B*. Previous animal studies showed high *SFRP2* expression during cardiogenesis and regeneration with anti-fibrotic properties, specifically in post-injury epicardium^25,31,54^. However, *SFRP5* has not been seen in animal studies and may be more relevant in humans. Studies found that *SFRP5* was inversely proportional to cardiovascular disease risk factors, positively correlated with faster recovery after MI and seen to protect against re-perfusion injury^26,55,56^. Although the local targets of these WNT proteins may be unknown, evidence suggests that *WNT2B* may increase proliferation in cardiomyocytes and fibroblasts^27,57^ and that restoring these elements of WNT signaling may be key to adult heart regeneration. Other vital epicardial ingredients involve extracellular matrix remodeling, proliferation and survival of myocardial tissue driven by *TGFB3*, *BMP3*, *RSPO1* (ref. 28) and a variety of epicardial-specific collagens, such as *COL11A1* (ref. 58). Lastly, we demonstrated that hESC-EPIs contain many of these ingredients and have proven effectiveness in animal model grafts^17^, giving confidence in this recipe, and that bringing fetal programs back into adult epicardium is a viable strategy for adult human heart regeneration.

A surprising result was the focus of adult epicardium on response to immune and external stimuli, which may be an undiscovered age-associated element of normal epicardial aging. This observation adds complexity and further weight to proper understanding of immune response regulation in cardiac regeneration as noted in experimental evidence in mice where a rapid transient immune response is key for proper regeneration^11^. This disparity between fetal and adult response programs places the epicardium further still as a key mediator of the immune response in cardiac regeneration with a coordinated age-associated upregulation of genes. Further still, this suggests that aging may elevate epicardial immuno-sensitivity as opposed to elevating the stimulatory abilities of immune cells. Lastly, our analysis also suggests a component of programmed aging that governs the loss of pro-regenerative functions with upregulated genes, such as *PLA2G2A*^59^ or *TNFSF14* (ref. 60). These genes have been implicated in inhibiting tumor angiogenesis and migration, which opens a discussion on what elements require deactivation as well as reactivation to produce a pro-regenerative epicardium.

It is important to note that our study did not capture adult hearts from a diseased population but, instead, focussed on the healthy state. Therefore, we could not compare the active fetal epicardium to injury-reactivated epicardium. We consider that these fetal programs could also become expressed in the injury-reactivated adult epicardium. For example, *SFRP2* and *SLIT3* are expressed in adult mice after injury^31^. Indeed, one experiment in neonatal mice revealed an increase in *RSPO1* in the regenerative P1 but not in non-regenerative P7 hearts after MI^12^. This forms one independent validation of healthy-state adults as a model of non-regenerative epicardium in humans, as *RSPO1* was also decreased in our adult epicardium. In our analysis, we grouped epicardial cells from multiple heart regions where there may be region-specific cellular compositions^1^. However, this is unlikely to affect the main biological comparison. On a final note, it is incorrect to assume that the entire regenerative capacity of the heart rests upon the active epicardium; other cells also play a major role in regeneration. Addressing this comprehensively is beyond the scope of this study. However, our integrated dataset may also be used for future tissue-targeted and organ-wide studies on the age-associated changes in the human heart.

The next step for clinical translation is to disentangle the gene networks that regulate adult and fetal epicardial states. In doing so, we might identify the molecular switches required to revert the adult epicardium into a fetal state and restore these key pathways. Finally, because we have detailed both active and inactive states of the human epicardium, benchmarked cross-species epicardial markers in humans and shown that stem-cell-derived epicardium contains regenerative epicardial programs, this study serves as a valuable roadmap toward reactivating the adult epicardium and promoting heart regeneration in adult humans.

## Methods

### Adult data collection

The unique molecular identifier (UMI) counts matrix for 486,134 adult heart cells or nuclei was acquired from the Heart Cell Atlas accessing the full version of the h5ad formatted dataset^22^. We then subset this matrix to retain only the six donors (D2–D7) with at least three cells annotated as ‘meso’ in the available ‘cell_state’ metadata, leaving 190,515 nuclei or cells.

### Fetal sample collection

Fetuses were obtained after elective termination of pregnancy with full consent (approved by the ethics committee of NHS East of England LREC no. 96/085) and stored overnight in Hibernate-A Medium (Gibco) at 4 °C. The next day after collection, the apex and base of each heart was dissected and dissociated^61^. In brief, tissue was dissociated using 6.6 mg ml^-1^ of *Bacillus licheniformis* protease, 5mM CaCl_2_ and 20 U ml^-1^ of DNase I, where the mixture was triturated on ice for 20 seconds every 5 minutes until clumps of tissue were no longer visible. The digestion was stopped with ice-cold 10% FBS in PBS. For sample F5, the apex and peeled epicardium was incubated with Liberase for 30 minutes, followed by washes. Cells were then washed with 10% FBS, resuspended in 1 ml of PBS and viability assessed using Trypan blue. Cells were submitted for 10x library preparation for 3′ single-cell sequencing on a NovaSeq 6000 (Illumina) using V3 chemistry at the Cancer Research UK (CRUK) Cambridge Institute. Sample F5 was collected as a pilot sample and prepared separately from the other fetal samples, and only the apex attached to a careful epicardial peeling was taken and dissociated. Sample F5 was sequenced independently at the Sanger Institute using HiSeq 4000 (Illumina).

### hESC-EPI differentiation and collection

Differentiation of epicardium was carried out according to our previously published protocols^47^. In brief, H9-hESCs (WiCell) were initially differentiated into lateral plate mesoderm (LM) in the presence of *FGF2* and *BMP4*. The LM is then exposed to *WNT3A*, *BMP4* and retinoic acid, resulting in hESC-EPIs after 8–9 days. Cells were harvested on days 1, 2, 3, 4, 8 and 9 of differentiation after the LM stage by re-suspension in PBS. Samples were submitted for 10x library preparation for 3′ single-cell sequencing at the CRUK Cambridge institute.

### RNA-seq pre-processing and processing

For fetal samples F1, F2, F3, F4, F6 and F7, demultiplexing, cell calling, alignment and counts matrix generation were carried out using Cell Ranger version 6.1. For our pilot sample F5, an earlier version of Cell Ranger was used. For the hESC-EPI samples, Cell Ranger version 3.02 was used. All samples were aligned against the human reference genome GRCh38 using default parameters. After the counts matrices were generated, all fetal samples were treated the same. Poor-quality cells were removed from the read counts matrices in R, retaining only cells with a depth of between 1,000 and 15,000 UMIs, expression of over 400 genes or fraction of mitochondrial genes under 15%. These thresholds were chosen following the boundaries of the adult dataset. Doublets in fetal datasets were called using Scrublet^62^ on the UMI matrices after alignment, with an expected doublet detection rate of 0.06. Erythrocyte contamination was seen in fetal samples, and erythrocytecontaining barcodes were identified using a two-compartment Gaussian mixture mode on the mean expression of hemoglobin genes in each sample. Fetal samples were then integrated and clustered. Clusters containing over 50% of the erythrocyte compartment were removed (Extended Data Fig. 1). For adult samples, pre-processing steps were not repeated as they were carried out before downloading the data. Finally, genes not expressed in any cell were removed from further analysis, leaving 27,956 gene features.

### Stratified sampling of datasets

Adult data were subsampled across mixed stratifications of ‘cell_state’, ‘donor’ and ‘cell_source’ annotations after data acquisition before any analysis. To do this, we built an algorithm using a single parameter *x* to control the sampling rate across *K* clusters of size *N_K_*, where *x* is the approximate sample size to take from each cluster *K*. Iteratively, our algorithm randomly sampled cells from each donor in each cluster *K* without replacement until the number of cells in the newly sampled cluster, *k*, exceeded *x* (*n_k_*>*x*). For cases where *N_k_*<*x*, the number of sampled cells is equal to the cluster size (*n_k_* = *N_K_*), effectively sampling all available cells and donors. This method was chosen to maximize minority cluster representation in the combined dataset while reducing source and donor biases. In the adult data, we aimed to subsample each ‘cell_state’ to the total number of epicardial cells (*n* = 717) in a two-stage sampling-stratified strategy. First, adult cells were sampled from ‘cell_state’ evenly distributed across ‘donor’ until each ‘cell_state’ consisted of at least the number of ‘Meso’-labeled nuclei (*x_cells_* = 597). Second and similarly, adult nuclei were sampled from ‘cell_state’ evenly distributed across ‘donor’ until 597 nuclei were sampled from each ‘cell_state’ annotation (*x_nuclei_* = 597). Finally, these newly sampled cells and nuclei were combined, and then the barcodes were re-sampled to the size of all ‘Meso’-annotated barcodes in the ‘cell_state’ annotation (Meso-labeled adult barcodes, *x_adult_*=717) to create evenly distributed groups of cell source, donor and cell type with a maximum similar cell type quantity to the number of epicardial cells (Extended Data Fig. 1).

To sample cells from the fetal datasets before integration, we first created new cell type stratifications. To do this, fetal samples were integrated using Seurat’s RPCA pipeline^63^. Following the established vignette, each fetal sample was log-transformed and scaled before integrating. After PCA, nearest neighboring cells were calculated using integrated distances, and fetal cells were clustered using the Louvain method of community detection (cells = 47,473, neighbors = 20, resolution = 0.5). This resulted in 21 cluster-based stratifications that were then sampled evenly across donors to achieve the size of the putative epicardial cell population identified using canonical epicardial markers (fetal epicardial cells, *x_fetal_*= 1,598) (Extended Data Fig. 1). The clustering parameters were chosen such that subsequent sampling of the number of epicardial cells from each cluster will result in a balance of adult and fetal cells in the sampled dataset (adult cells = 29,779, fetal cells = 30,889). We included all fetal epicardial cells with the aim of retaining the maximum information available for this uncommon cell population.

### Integration of adult and fetal data

Raw UMI counts matrices of subsampled adult and fetal datasets were combined following Seurat’s RPCA integration pipeline^63^. In brief, we defined 24 new integration groups within the dataset combining the unique combinations of ‘cell_source’ and ‘donor’ annotations. Each integration group was then log-transformed and scaled individually, and variable features were identified, followed by PCA. We selected 2,806 anchor genes for integration as found to be variable in at least 25% of the integration groups. Additionally, to perform our integration, we defined a hierarchical sample tree for integrating these 24 groups, which prioritized (1) the donor-matched integration of adult nuclei into adult cells and fetal base into apex, followed by (2) integration between fetal donors and then (3) integration of all adult data into fetal data (Extended Data Fig. 1). We chose this method as our samples consisted of different sequencing samples or data sources from the same donor (four sources: nuclei, cell, apex and base). Clustering across the integrated dataset was performed using the Louvain algorithm in Seurat, giving ten distinct cell types (resolution = 0.1, *k* neighbors = 20). Each cell type cluster was then similarly and separately subclustered, giving 56 high-resolution clusters. Then, to represent the data over multiple cell type and state granularities, we aggregated these high-resolution clusters together hierarchically by joining biologically similar cell types together at decreasing resolutions until we arrived at the initial low-resolution clustering (Extended Data Fig. 2).

### Epicardial markers and epicardial markers library

A stage-separated epicardial marker analysis was performed in parallel using the fetal and adult datasets after annotation with the newly defined stage-independent clusters in Seurat with a one-cluster-versus-all strategy on log-transformed counts across clusters of resolutions 1 and 2. We used Wilcoxon rank-sum tests and applied thresholds of *P* <1× 10^-10^ and absolute log_2_ fold change > 0.5 throughout the study where significance scores were adjusted for multiple comparisons using Bonferroni correction. In Fig. 1, we reported resolution 1 results comparing the broader gene expression of epicardial cells with all other cell types without resolving epicardial subclusters. Additionally, two lists of unwanted variation genes were created using differential expression analyses. The first list compared all adult cells and all fetal cells to capture a non-specific age-associated gene list of six adult genes and 92 fetal genes. The second list compared adult cells and adult nuclei to capture the genes associated with the nuclei ‘cell_source’, identifying 69 nuclei genes. These genes were omitted from the results and resulted in 633 and 724 epicardial genes at clustering resolutions 1 and 2 (unique from epicardial subclusters), respectively. The 724 resolution 2 marker genes were then scored using the number of gene-positive cells in each group of fetal-epicardial, adult-epicardial or non-epicardial cells where, for each gene and for each group, we identified its precision (analogous to specificity) determined by the fraction of positive cells that belong to the group; recall (analogous to sensitivity), as the fraction of group cells that were positive; and the *F*-score, calculated as the harmonic mean between recall and precision (*F_1_* =2 × (recall × precision) / (recall + precision)). To further enrich this library distinguishing between epicardial cell types ‘FB-like’ and ‘Mesothelial’, we restricted the dataset to *UPK3B*^+^ cells as *UPK3B*. This was necessary as there were no clear single markers that separated the EPDCs from other cells of the heart as well as epicardial cells. Using *UPK3B*^+^ cells only, we then re-calculated recall, precision and *F*-scores of the epicardial genes using the groups of ‘FB-like’ and ‘Mesothelial’. This generated a list of differential markers within the *UPK3B*^+^ population of cells to discern between potential EPDCs and mesothelial epicardium. Then, for each group of ‘Fetal’, ‘Shared’, ‘Adult’, ‘Mesothelial’ or ‘EPDC’ cells, genes were re-ordered by subtracting the *F*-score for other groups from the *F*-score for all groups. This resulted in a per-group ordering of genes by selectivity.

### Ligand receptor analysis with CellPhoneDB

The combined adult and fetal counts matrix was transformed into counts per million (CPM) as recommended and subsequently log_2_-transformed in the CellPhoneDB statistical analysis pipeline using the curated interactions database (version 2.0)^64^. For this analysis, the adult epicardial subclusters of Adult_Epicardium_Proliferating and Adult_Epicardium_FB-like were omitted as there were fewer than three cells in each (*n* = 0 and 2, respectively). The numbers of significant interactions for the remaining epicardial clusters were then counted in either adult or fetal stages (*P* < 0.05) and visualized in an ordered heat map. The results from CellPhoneDB were filtered in R, retaining only epicardial-specific markers identified in the parallel marker analysis. This was further filtered for putative secreted protein-coding genes using the Human Protein Atlas as a reference.

### Gene–gene co-occurrence and epicardial gene module construction

Cells annotated as epicardium after clustering were isolated in a new matrix, and a set of epicardial features for clustering was selected using differential expression analysis between adult and fetal epicardial cells (Wilcoxon rank-sum test; *P* <1× 10^-10^, log_2_ fold change > 0.5) (Supplementary Table 10). After removing the previously identified control genes, the epicardial matrix of 1,912 cells across 1,594 genes was binarized, counting positive expression as a value of at least 1. For gene module construction, this matrix was further subset to omit nuclei barcodes, as gene clustering was shown to be affected largely by their expression in nuclei or cells, giving a binary matrix of 1,315 cells and 1,594 genes. However, nuclei were added back into the matrix after module construction, and we observed that nuclei largely retained gene module patterns, independently validating our results. We implemented the approach by Qiu (ref. 65) in R to cluster gene-dropout patterns. In brief, we calculated the co-occurrence of each gene pair across all cells with a modified chi-square statistic. Then, for each gene pair, all chi-square statistics below a given threshold were discarded to retain only the high-scoring gene–gene pairs. This threshold was calculated using random permutations of the data. An undirected graph of highly concerted genes was then formed from the remaining gene pairs, weighted by the chi-square statistics and adjusted by a Jaccard index. Finally, this gene–gene graph was clustered using a conservative Louvain method for community detection, removing all clusters with fewer than 20 genes. Genes in resulting gene modules were ordered by their mean chi-square value with other genes in the module, ranking the genes by pattern specificity. New gene module commitment features for all epicardial cells, including nuclei, were calculated by the mean of binarized expression of each gene module, and a PCA was carried out. We represented components 2 and 4, as component 1 was technical noise, correlating with library size and source (Kendall rank correlation, *P* < 0.05) (Fig. 3b and Extended Data Fig. 3), whereas components 2 and 4 correlated with cell type and age and were orthogonally represented in the PCA. The Louvain method for community detection was used to generate epicardial states, and states were ordered by age using their mean of PCA component 2, which highly correlated with the sample-ranked age of each cell (Kendall’s tau = –0.67, *P*< 0.01, *n* = 1,912). Gene set overrepresentation analysis of Gene Ontology terms across gene modules was carried out using the R package gprofiler2 (ref. 66) against a background of all expressed genes in epicardial cells.

### hESC-EPI analysis and classification

After pre-processing of the hESC-EPI data, 300 cells were randomly sampled in silico from each timepoint. The sampled dataset of 1,800 cells was log-transformed, subjected to PCA and projected into two UMAP dimensions using Seurat. The Louvain method of community detection was then used to cluster cells, and the clusters were annotated into either of previously identified lineages based on marker expression. To classify the hESC-EPIs, a random forest classification model was trained on the in vivo dataset to discern among 34 cell types in either adult or fetal stages from clustering resolution 3 (Extended Data Fig. 4). In training and testing the random forest classifier, raw UMI matrices were processed in an experiment-independent manner, and cells were adjusted for library size using CPM and transformed using log_2_ with a pseudocount of 1. Features selected for the model were the top 50 differentially expressed marker genes from each cluster in the resolution 2 clusters from the stage-separated analysis (Supplementary Table 2), giving a total of 1,445 unique features. The model was constructed on a sample of 33% of cells and assessed using six-fold cross-validation and independently validated on the remaining 66% of cells (Extended Data Fig. 5). The sampled hESC-EPI dataset of 1,800 cells was then predicted using the model trained on the 33% cell fraction.

### Immunofluorescent staining

Fetal hearts were collected from donors BRC2281 and BRC2375, aged 9 weeks and 10 weeks, 3 days, respectively, and were dissected and fixed in 4% paraformaldehyde (Alfa Aesar) overnight with gentle rocking at 4 °C. Then, cryoprotection was done in 30% sucrose (Sigma/Merck) for a further 24 hours at 4°C. Tissues were embedded in OCT (Sakura Tek) and frozen on dry ice. Then, 10-μm sections were cut serially using a Leica cryostat. Sections were air dried for at least 10 minutes before storage at −80 °C. Immunofluorescent staining was carried out by thawing the slides for 10 minutes and rehydrating with tris-buffered saline (TBS) for a further 10 minutes at room temperature. The tissue was permeabilized for 10 minutes in a permeabilization buffer made up of 0.25% saponin in TBS, followed by a 5-minute wash with 0.2% Tween 20 in TBS. A 0.3 M glycine in antibody dilution blocking buffer was applied for 1 hour. Then, this solution was decanted, and the primary antibody solution was applied overnight at 4 °C (all primary antibodies were used at a dilution of 1:100). The next day, the tissue was subjected to three 5-minute washes, and the secondary antibody solution was applied (all secondary antibodies were used at a dilution of 1:1,000) for 1 hour at room temperature. The secondary antibody solution was washed for 5 minutes a following two times, and a DAPI solution was added at a diluation of 1:2,000 in TBS for 15 minutes at room temperature. This was washed for 5 minutes. To finish the process, VectaShield was applied and a coverslip attached. Each tissue was left for at least 2 hours before imaging. The primary antibodies used in this study for immunofluorescent imaging included: *UPK3B*, PA552696 (Themo Fisher Scientific); *MSLN*, sc33672 (Santa Cruz Biotechnology); *KRT19*, sc6278 (Santa Cruz Biotechnology); *POSTN*, MAB3548 (R&D Systems); *DCN*, AF143 (R&D Systems); *PRG4*, MABT400 (Sigma-Aldrich); and *TM4SF1*, MAB8164 (R&D Systems). The secondary antibodies used included: Alexa Fluor 488 donkey anti-rabbit A21206 (Invitrogen) for *UPK3B*; Alexa Fluor 647 goat anti-mouse A21240 (Invitrogen) for MSLN, KRT19 and TM4SF1; Alexa Fluor 568 goat anti-rat A11077 (Invitrogen) for POSTN; Alexa Fluor 647 chicken anti-mouse A21463 (Invitrogen) for PRG4; and Alexa Fluor 568 donkey anti-goat A11057 (Invitrogen) for DCN. A hydrophobic pen was used throughout the staining process to surround the tissue.

### Visium slide and library preparation for spatial transcriptomics and Cell2Location

A single heart, 9 weeks, 4 days post-conception, was frozen and embedded in OCT medium using a dry-ice-cooled bath of isopentane. OCT-embedded samples were sectioned using a cryostat (Leica, CX3050S) and cut at 10 μm. RNA integrity number (fresh-frozen samples) was obtained using an Agilent 2100 Bioanalyzer. The Tissue Optimization protocol from 10x Genomics was performed to obtain a permeabilization time of 35 minutes, and the Visium Spatial Gene Expression experiment was performed as per the manufacturer’s protocol (10x Genomics). Hematoxylin and eosin (H&E)-stained Visium Gene Expression slides were imaged at ×40 on a Hamamatsu NanoZoomer S60. After transcript capture, Visium Library Preparation Protocol from 10x Genomics was performed. The cDNA library was diluted to a final concentration of 2.25 nM (200 μl volume) and sequenced on 2× SP flow cells of Illumina NovaSeq 6000. Space Ranger (version 1.1.0, 10x Genomics) was used for the read-mapping to the human reference genome (GRCh38) with default parameters. Anatomical microstructures were manually annotated using the paired histology H&E image. To map clusters onto Visium results, we used Cell2Location^67^. In brief, Cell2Location first estimates reference signatures of cell types obtained from the scRNA-seq data using a negative binomial regression model. Then, the abundance of each cell type is calculated in each Visium spot by decomposing spot mRNA counts using the cell type signatures. A spot resolution hyperparameter was estimated using H&E-stained images of the Visium slides, resulting in 20 cells per spot for parameterizing the Cell2Location pipeline.

### Ethics statement

Collection of human fetuses for this study from anonymous female donors was approved by NHS East of England under LREC no. 96/085. Full informed consent was given by all donors after elective termination of pregnancy. Donors were made aware of the possible use of donated fetuses, and no financial compensation was given. Donors were free to withdraw consent at any time.

### Reporting summary

Further information on research design is available in the Nature Portfolio Reporting Summary linked to this article.

## Extended Data

## Supplementary Material

Source Data Fig.4

Supplementary Table 1

Supplementary Table 2

Supplementary Table 3

Supplementary Table 4

Supplementary Table 5

Supplementary Table 6

Supplementary Table 7

Supplementary Table 8

Supplementary Table 9

## Data Availability

Both raw and processed RNA sequencing data generated during this study can be found in the Gene Expression Omnibus using the accession identifiers GSE216019 (fetal scRNA-seq data) and GSE216177 (hESC-EPI differentiation). Matrices of adult heart scRNA-seq data are available from the Heart Cell Atlas^27^ and can be accessed at https://www.heartcel-latlas.org/#DataSources. Specifically, the adult data file accessed was downloaded here: https://cellgeni.cog.sanger.ac.uk/heartcellatlas/data/global_raw.h5ad. Finally, the integrated data combining both adult and fetal data may be explored interactively at http://sinha.stem-cells.cam.ac.uk/. The human reference genome (GRCh38) is available at https://cf.10xgenomics.com/supp/cell-exp/refdata-gex-GRCh38-2020-A.tar.gz.

## Code availability

The R code and reports generated during this analysis are freely available from GitHub (https://github.com/Hindrance/Adult_Foetal_Epicardial_Comparison_2022).

## References

[1] Risebro CA, Vieira JM, Klotz L, Riley PR (2015). Characterisation of the human embryonic and foetal epicardium during heart development. Development.

[2] Krainock M (2016). Epicardial epithelial-to-mesenchymal transition in heart development and disease. J Clin Med.

[3] Dettman RW, Denetclaw W, Ordahl CP, Bristow J (1998). Common epicardial origin of coronary vascular smooth muscle, perivascular fibroblasts, and intermyocardial fibroblasts in the avian heart. Dev Biol.

[4] Katz TC (2012). Distinct compartments of the proepicardial organ give rise to coronary vascular endothelial cells. Dev Cell.

[5] Carmona R (2010). The embryonic epicardium: an essential element of cardiac development. J Cell Mol Med.

[6] Lepilina A (2006). A dynamic epicardial injury response supports progenitor cell activity during zebrafish heart regeneration. Cell.

[7] González-Rosa JM, Martín V, Peralta M, Torres M, Mercader N (2011). Extensive scar formation and regression during heart regeneration after cryoinjury in zebrafish. Development.

[8] Bryant DM (2015). A systematic analysis of neonatal mouse heart regeneration after apical resection. J Mol Cell Cardiol.

[9] Haubner BJ (2016). Functional recovery of a human neonatal heart after severe myocardial infarction. Circ Res.

[10] Ye L (2018). Early regenerative capacity in the porcine heart. Circulation.

[11] Wang Z (2019). Mechanistic basis of neonatal heart regeneration revealed by transcriptome and histone modification profiling. Proc Natl Acad Sci USA.

[12] Wang Z (2020). Cell-type-specific gene regulatory networks underlying murine neonatal heart regeneration at single-cell resolution. Cell Rep.

[13] Zhou B (2011). Adult mouse epicardium modulates myocardial injury by secreting paracrine factors. J Clin Invest.

[14] Smart N (2012). Myocardial regeneration: expanding the repertoire of thymosin β4 in the ischemic heart. Ann N Y Acad Sci.

[15] Virag JA (2007). Fibroblast growth factor-2 regulates myocardial infarct repair: effects on cell proliferation, scar contraction, and ventricular function. Am J Pathol.

[16] Villa Del Campo C (2021). Regenerative potential of epicardium-derived extracellular vesicles mediated by conserved miRNA transfer. Cardiovasc Res.

[17] Bargehr J (2019). Epicardial cells derived from human embryonic stem cells augment cardiomyocyte-driven heart regeneration. Nat Biotechnol.

[18] Braitsch CM, Combs MD, Quaggin SE, Yutzey KE (2012). Pod1/Tcf21 is regulated by retinoic acid signaling and inhibits differentiation of epicardium-derived cells into smooth muscle in the developing heart. Dev Biol.

[19] Mantri M (2021). Spatiotemporal single-cell RNA sequencing of developing chicken hearts identifies interplay between cellular differentiation and morphogenesis. Nat Commun.

[20] Weinberger M, Simões FC, Patient R, Sauka-Spengler T, Riley PR (2020). Functional heterogeneity within the developing zebrafish epicardium. Dev Cell.

[21] Gambardella L (2019). BNC1 regulates cell heterogeneity in human pluripotent stem cell-derived epicardium. Development.

[22] Litviňuková M (2020). Cells of the adult human heart. Nature.

[23] Lupu I-E, Redpath AN, Smart N (2020). Spatiotemporal analysis reveals overlap of key proepicardial markers in the developing murine heart. Stem Cell Rep.

[24] Suryawanshi H (2020). Cell atlas of the foetal human heart and implications for autoimmune-mediated congenital heart block. Cardiovasc Res.

[25] Wu Y (2020). Multiple roles of sFRP2 in cardiac development and cardiovascular disease. Int J Biol Sci.

[26] Nakamura K (2016). Secreted frizzled-related protein 5 diminishes cardiac inflammation and protects the heart from ischemia/ reperfusion injury. J Biol Chem.

[27] Peng X (2020). Wnt2bb induces cardiomyocyte proliferation in zebrafish hearts via the jnk1/c-jun/creb1 pathway. Front Cell Dev Biol.

[28] Li G (2019). Single cell expression analysis reveals anatomical and cell cycle-dependent transcriptional shifts during heart development. Development.

[29] Khosravi F, Ahmadvand N, Bellusci S, Sauer H (2021). The multifunctional contribution of FGF signaling to cardiac development, homeostasis, disease and repair. Front Cell Dev Biol.

[30] Barker H (2017). Role of carbonic anhydrases in skin wound healing. Exp Mol Med.

[31] Hesse J (2021). Single-cell transcriptomics defines heterogeneity of epicardial cells and fibroblasts within the infarcted murine heart. eLife.

[32] Quijada P (2021). Coordination of endothelial cell positioning and fate specification by the epicardium. Nat Commun.

[33] Alissa EM, Al-Salmi MM, Alama NA, Ferns GA (2016). Role of omentin-1 and C-reactive protein in obese subjects with subclinical inflammation. J Clin Transl Endocrinol.

[34] Cui Y (2019). Single-cell transcriptome analysis maps the developmental track of the human heart. Cell Rep.

[35] Park DSJ (2018). Human pericardial proteoglycan 4 (lubricin): implications for postcardiotomy intrathoracic adhesion formation. J Thorac Cardiovasc Surg.

[36] Hamm MJ, Kirchmaier BC, Herzog W (2016). Sema3d controls collective endothelial cell migration by distinct mechanisms via nrp1 and PlxnD1. J Cell Biol.

[37] Bonet F, Inácio JM, Bover O, Añez SB, Belo JA (2022). CCBE1 in cardiac development and disease. Front Genet.

[38] Kang J (2020). The emerging role of EGFL6 in angiogenesis and tumor progression. Int J Med Sci.

[39] Kolluri A, Ho M (2019). The role of glypican-3 in regulating Wnt, YAP, and hedgehog in liver cancer. Front Oncol.

[40] Rossignol M, Gagnon ML, Klagsbrun M (2000). Genomic organization of human neuropilin-1 and neuropilin-2 genes: identification and distribution of splice variants and soluble isoforms. Genomics.

[41] Roy S (2017). Multifaceted role of neuropilins in the immune system: potential targets for immunotherapy. Front Immunol.

[42] Descamps B (2012). Frizzled 4 regulates arterial network organization through noncanonical Wnt/planar cell polarity signaling. Circ Res.

[43] Molin DG (2003). Expression patterns of Tgfβ1-3 associate with myocardialisation of the outflow tract and the development of the epicardium and the fibrous heart skeleton. Dev Dyn.

[44] Doetschman T (2012). Transforming growth factor beta signaling in adult cardiovascular diseases and repair. Cell Tissue Res.

[45] Xue K (2019). The role and mechanism of transforming growth factor beta 3 in human myocardial infarction-induced myocardial fibrosis. J Cell Mol Med.

[46] Shuvalova YA (2015). The association of PLA2G2A single nucleotide polymorphisms with type IIa secretory phospholipase A2 level but not its activity in patients with stable coronary heart disease. Gene.

[47] Iyer D (2015). Robust derivation of epicardium and its differentiated smooth muscle cell progeny from human pluripotent stem cells. Development.

[48] Moerkamp AT (2016). Human fetal and adult epicardial-derived cells: a novel model to study their activation. Stem Cell Res Ther.

[49] Winter EM (2007). Preservation of left ventricular function and attenuation of remodeling after transplantation of human epicardium-derived cells into the infarcted mouse heart. Circulation.

[50] He L (2017). Preexisting endothelial cells mediate cardiac neovascularization after injury. J Clin Invest.

[51] Paul JD (2013). SLIT3-ROBO4 activation promotes vascular network formation in human engineered tissue and angiogenesis in vivo. J Mol Cell Cardiol.

[52] Cavallero S (2015). CXCL12 signaling is essential for maturation of the ventricular coronary endothelial plexus and establishment of functional coronary circulation. Dev Cell.

[53] Schneider C (2020). Primate heart regeneration via migration and fibroblast repulsion by human heart progenitors. https://www.biorxiv.org/content/10.1101/2020.07.03.183798v1.full.

[54] He W (2010). Exogenously administered secreted frizzled related protein 2 (Sfrp2) reduces fibrosis and improves cardiac function in a rat model of myocardial infarction. Proc Natl Acad Sci USA.

[55] Carstensen-Kirberg M (2017). Inverse associations between serum levels of secreted frizzled-related protein-5 (SFRP5) and multiple cardiometabolic risk factors: KORA F4 study. Cardiovasc Diabetol.

[56] Du Y (2019). High serum secreted frizzled-related protein 5 levels associates with early improvement of cardiac function following ST-segment elevation myocardial infarction treated by primary percutaneous coronary intervention. J Atheroscler Thromb.

[57] Liu S (2021). Yap promotes noncanonical Wnt signals from cardiomyocytes for heart regeneration. Circ Res.

[58] Lincoln J, Florer JB, Deutsch GH, Wenstrup RJ, Yutzey KE (2006). ColVa1 and ColXIa1 are required for myocardial morphogenesis and heart valve development. Dev Dyn.

[59] Ganesan K (2008). Inhibition of gastric cancer invasion and metastasis by PLA2G2A, a novel β-catenin/TCF target gene. Cancer Res.

[60] He B (2018). Vascular targeting of LIGHT normalizes blood vessels in primary brain cancer and induces intratumoural high endothelial venules. J Pathol.

[61] Menon R (2018). Single-cell analysis of progenitor cell dynamics and lineage specification in the human fetal kidney. Development.

[62] Wolock SL, Lopez R, Klein AM (2019). Scrublet: computational identification of cell doublets in single-cell transcriptomic data. Cell Syst.

[63] Stuart T (2019). Comprehensive integration of single-cell data. Cell.

[64] Efremova M, Vento-Tormo M, Teichmann SA, Vento-Tormo R (2020). CellPhoneDB: inferring cell-cell communication from combined expression of multi-subunit ligand-receptor complexes. Nat Protoc.

[65] Qiu P (2020). Embracing the dropouts in single-cell RNA-seq analysis. Nat Commun.

[66] Raudvere U (2019). g:Profiler: a web server for functional enrichment analysis and conversions of gene lists (2019 update). Nucleic Acids Res.

[67] Kleshchevnikov V (2022). Cell2location maps fine-grained cell types in spatial transcriptomics. Nat Biotechnol.

