# Author Correction: A single-cell comparison of adult and fetal human epicardium defines the age-associated changes in epicardial activity

**DOI:** 10.1038/s44161-023-00269-z

**Published:** 2023-04-11

**Authors:** Vincent R. Knight-Schrijver, Hongorzul Davaapil, Semih Bayraktar, Alexander D. B. Ross, Kazumasa Kanemaru, James Cranley, Monika Dabrowska, Minal Patel, Krzysztof Polanski, Xiaoling He, Ludovic Vallier, Sarah Teichmann, Laure Gambardella, Sanjay Sinha

**Affiliations:** 1 Wellcome-MRC Cambridge Stem Cell Institute, Jeffrey Cheah Biomedical Centre, Cambridge Biomedical Campus, University of Cambridge, Cambridge, UK; 2 Department of Paediatrics, University of Cambridge, Cambridge, UK; 3 Department of Medical Genetics, University of Cambridge, Cambridge, UK; 4 Wellcome Sanger Institute, Wellcome Genome Campus, Cambridge, UK; 5 John van Geest Centre for Brain Repair, Cambridge University, Cambridge, UK; 6 Berlin Institute of Health (BIH), BIH Centre for Regenerative Therapies (BCRT), Charité - Universitätsmedizin, Berlin, Germany; 7 Max Planck Institute for Molecular Genetics, Berlin, Germany; 8 Cavendish Laboratory, Department of Physics, University of Cambridge, Cambridge, UK

**Keywords:** Heart development, Ageing, Stem-cell research, Regeneration, RNA sequencing

Correction to: *Nature Cardiovascular Research* 10.1038/s44161-022-00183-w. Published online 21 December 2022.

In the version of this article initially published, in the first sentence of the Results, in the first sentence of the Methods subsection “Visium slide and library preparation for spatial transcriptomics and Cell2Location,” and in the legend of Extended Data Fig. 7, fetal ages were incorrectly described as “weeks post-conception” rather than as gestational age. The errors have been corrected in the HTML and PDF versions of the article.

